# Widespread fear of dengue transmission but poor practices of dengue prevention: A study in the slums of Delhi, India

**DOI:** 10.1371/journal.pone.0171543

**Published:** 2017-02-10

**Authors:** Éric Daudé, Sumit Mazumdar, Vandana Solanki

**Affiliations:** 1 The National Center for Scientific Research, Center for Social Sciences and Humanities, Delhi, India; 2 Institute of Public Health, Kalyani, West Bengal, India; Fundacao Oswaldo Cruz, BRAZIL

## Abstract

**Background:**

This study has been conducted to throw light on the knowledge and practices related to dengue fever among the poor population living in Delhi’s slums.

**Materials:**

A household survey was conducted in 2013 among 3,350 households. The households were stratified by a number of variables related to socio-economic status and health events such as hospitalisation. The data collection was completed through face-to-face interviews conducted with the help of 25 field investigators.

**Results:**

About 8% of the households had at least one diagnosed dengue case. In comparison to the population surveyed, teenagers (15–19 years) and adults (30–34 years) were more affected whereas children under four years of age were underrepresented. Housewives are more affected by dengue (24%) compared to their share of the population surveyed (17%). Despite the fact that 77% of the respondents are worried about mosquitoes, only 43% of them monitor environment to avoid the presence of breeding sites.

**Conclusion:**

One cannot exclude the possibility that though young children under the age of four years are exposed to the virus, either their cases were asymptomatic or family members infected during this period had potentially more serious symptoms leading to hospitalisation. This result could thus be explained by budget-related health choices made by this population which do not favour small children. Educational programs should target housewives to improve their impact, as they are the ones mostly responsible for water storage and cleanliness of the house and its neighbourhood. Even with a dengue experience and potentially an acute perception of the risk and its factors, a proper management of environmental conditions is lacking. This along with the fact that word-of-mouth is the main source of information quoted should be a message for municipality health workers to give door-to-door information on how to prevent breeding sites and dengue infection.

## Introduction

Dengue is an infectious viral disease transmitted by a domesticated mosquito, *Aedes aegypti*. There are supposedly 50 [1] to 400 million people [2] infected each year by dengue virus, developing various clinical spectrum of dengue infection that includes asymptomatic cases, a self-limited acute febrile syndrome and severe and fatal cases of haemorrhagic fever with shock. In tropical and sub-tropical countries where the disease is mostly prevalent, dengue becomes endemic and even hyper-endemic, leading to major public health problems. Several factors contribute to the spread of this disease in developing countries. A not-always-planned urban growth favours the presence of breeding sites for this synanthropic mosquito, which makes its control difficult [3]. The flow of migrants from neighbouring States and other countries introduces dengue virus and new serotypes [4]. Lack of information, education and communication regarding the vector and the disease are not conducive to changes in human behaviour [5]. Finally, weak public health services do not permit effective surveillance and control of the disease [6, 7].

Since 1967 [8], Delhi records dengue epidemics every 3–4 years i.e. 1970 [9], 1982 [10], 1988 [11], 1996 [12], 2003 [13], 2006 [14], 2010 [15], 2013 [16] and 2015 [17]. Data provided by the official surveillance system reports that the dengue outbreak of the year 2013 led to 5,574 hospitalisations and 8 deaths [17]. This is mainly due to the rapid growth of the city and the presence of several poor areas providing suitable conditions for breeding of *Aedes aegypti* [18]. Studies show that awareness of the disease and its symptoms are at medium levels in India [19–20] and an effective knowledge about the process of vector transmission and ways to prevent it are not consistently well understood [21]. These issues are certainly more acute in islands of urban poverty as it is difficult to maintain regular surveillance and control of the mosquito breeding sites [22]. It is also difficult to estimate the prevalence of dengue when sick people do not have access to health services and are not recorded in sentinel hospitals. In the absence of a vaccine, it is fundamental that people act against the disease and its local risk of transmission in an efficient and appropriate manner by ensuring vector control in their neighbourhood, notably by eliminating stagnant water within and around their homes in order to limit the presence of mosquitoes.

A large proportion, estimated to be close to 65% of dengue infection in Delhi, is asymptomatic, leading to insufficiency of vector and dengue control based on hospital records alone [23]. Yet, among the poor populations, in addition to these asymptomatic cases, we must add infected people with sub-clinical symptoms who will not visit a physician for a simple fever. To prevent dengue in these areas, it is then important to estimate and if necessary improve dengue-related knowledge, awareness and practices among the people. Despite frequent dengue epidemics occurring in Delhi, few studies have been conducted on the poor. This study assesses the patterns and differences in knowledge and practices related to transmission and prevention of dengue based on various indicators such as sex, literacy and age focusing on spatially and socio-economically poorer segments of the population residing in settlements known as Jhuggi-Jhompri (JJ) clusters.

## Materials and methods

**Fig 1:**
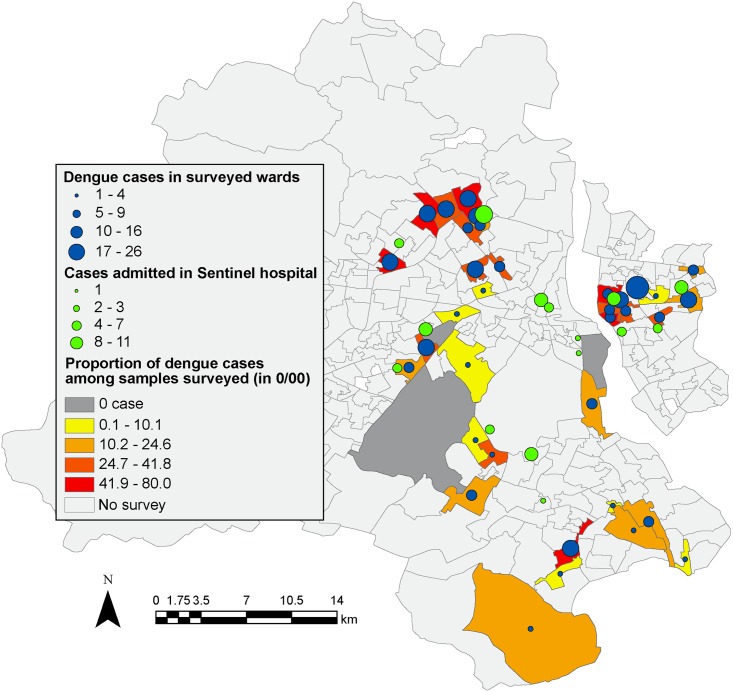
Map of the surveyed area. The colours of the wards (from yellow to red) represent the proportion (in thousands) of the number of dengue cases related to the surveyed population. Blue circles represent the total number of confirmed dengue cases in the survey. Green circles represent dengue cases that have been diagnosed in a sentinel hospital.

The data used in this paper is based on a household survey (S1 File) conducted between November 2013 and May 2014 among 3,350 families residing in low-income urban communities in Delhi (Fig 1), India, as part of a larger research project on impact evaluation of India’s national social health insurance scheme, Rashtriya Swasthya Bima Yojana (RSBY) [24].

RSBY is a targeted health insurance scheme offering cashless hospitalisation services in designated hospitals for officially identified poor households. Acknowledging the limitations of using secondary lists to identify poor households in Delhi, a screening survey of about 10,000 households was initiated to identify the urban poor. Considering the spatial distribution of poverty in urban areas instead of working at municipal ward level, it was decided to conduct the entire survey on a specific type of slums, locally known as Jhuggi Jhompri (JJ) settlements spread across all the districts in Delhi. It is pertinent to note that these JJ settlements are the most underdeveloped localities in Delhi with a high concentration of poor households and low coverage of civic amenities and health care facilities.

Lists of all JJ clusters for each of the 11 districts were collected from the Delhi Urban Shelter Improvement Board (DUSIB). In the subsequent stage, all these settlements in their respective districts were arranged according to their population and those with a population of 2,000 or more (N = 52), were considered for a screening survey—primarily conducted in order to screen insured and non-insured households (under RSBY). In each JJ settlement, 200 households were randomly sampled leading to 10,707 screened sample households across the 52 selected settlements. In the next stage, screened households were stratified based on socioeconomic characteristics and divided into groups based on the sample selection criteria for the main project viz. possession of health insurance (RSBY) cards, and experiences of hospitalisation among family members. Finaly about one-third households were sampled from each stratum using systematic random sampling which lead to 3,350 households (for details about sample method, see S2 File).

The head of household was the respondent for the household survey; in his or her absence, other members above 16 years of age and found knowledgeable were selected. Data collection was completed through face-to-face, pen-and-paper interviews conducted with the help of 25 field investigators. This data was entered in raw MS-Access 2010 format (Microsoft, Redmond, USA) and analysed with the Data Analysis and Statistical Software STATA 13.0 (StataCorp LP, Texas, USA). Results were recorded as frequencies (%) and standard deviation (SD). Chi-square test of independence was computed to determine independence between epidemiological status of the population and socio-demographic variables. The critical chi squared value is given for α = 0.05.

The dataset collected during the survey consists rich information on a number of demographic, socioeconomic, health status and health service use variables such as age, sex, education, caste, monthly per capita expenditure (MPCE) and health expenditure. In this paper, we focus on two domains, knowledge about dengue and preventive measures against the disease. Another point to note is that as there was one respondent per household and as the survey mainly focused on the adoption level of the national social health insurance scheme, it was not possible to interview all the people infected by dengue virus. It is important to note that neither did we conduct any biological confirmation of the interviewed people’s immunological status nor did we check the blood test results. These 263 interviewed respondents were recruited according to the answer ‘*Yes’* to the questions “*Did you get any blood tests done*?” followed by “*Were you informed/did you know the results of the tests*?” and answer ‘*dengue*” to the question “*What was the diagnosis*?” during the survey.

The study was conducted in compliance with “*Ethical Principles for Medical Research Involving Human Subjects*” as under the Helsinki declaration. The internal review committee at Institute for Human Development (IHD) was constituted to review, comment and approve all aspects pertaining to any research study, including research design, selection of respondents and questions asked. The committee is also mandated to review the ethical considerations such as informed consent, nature of questions and their sensitivity given the context and information collected for minors. The committee has researchers as its members from different disciplines, mainly from social sciences and liberal arts. One member is invited from legal field and a medical professional for projects related to health and nutrition is included.

For the present survey, informed oral consent has been obtained for each participant. It was not possible to obtain written consent given the low literacy level in the slums.

## Results

### Study setting, socio-demographics and health scenario

Characteristics of the studied participants are summarized in Table 1 for the total population (N = 18,192 inhabitants) and for households (N = 263) where at least one dengue case has been recorded. In fact, none of the households declared more than one diagnosed dengue case in the last 6 months.

**Table 1:** Socio-demographic profile of the survey population (N = 18,192) and for dengue cases (N = 263).

Socio-demographic characteristics	N (%) of respondents	N (%) of respondents diagnosed with dengue
*Total sample*	18,192 person	263 person
*Gender (χ*^*2*^*calc*. *= 1*.*16 < 3*.*84)*		
Male	9,661 (53.1%)	131 (49.8%)
Female	8,531 (46.9%)	132 (50.1%)
*Mean age (years) ± SD*	25.1 ± 17	25.9 ± 15
*Age distribution (years) (χ*^*2*^*calc*. *= 20*.*17 > 18*.*31)*		
0–4	1,416 (8%)	8 (3%)
5–9	1,938 (11%)	20 (8%)
10–14	2,226 (12%)	33 (13%)
15–19	2,539 (14%)	47 (18%)
20–24	2,255 (12%)	37 (14%)
25–29	1,556 (9%)	21 (8%)
30–34	1,194 (7%)	25 (10%)
35–39	1,152 (6%)	18 (7%)
40–44	1,017 (6%)	15 (6%)
45–49	904 (5%)	17 (6%)
>50	1,995 (11%)	22 (8%)
*Education level (χ*^*2*^*calc*. *= 10*.*13 < 14*.*07)*		
Above Graduation	33 (0.19%)	0 (0%)
Completed Graduation	429 (2.5%)	5 (2%)
Completed class Twelve	1,189 (7%)	16 (6%)
Completed class Tenth	2,150 (13%)	46 (18%)
Up to class Eight	3,440 (20%)	57 (22%)
Up to class Fifth	4,423 (26%)	54 (21%)
Literate but never gone to school	1,281 (8%)	17 (7%)
Illiterate	4,036 (24%)	60 (24%)
*Main occupation (χ*^*2*^ *calc*. *= 15*.*27 < 18*.*31)*		
Casual/daily wage labourer	2,835 (16%)	41 (16%)
Disabled/too Old/too Young	1,631 (9%)	11 (4%)
HH entrepreneur (tailoring/weaning)	211 (1%)	1 (0%)
Home-maker/household work (unpaid)	688 (4%)	10 (4%)
Housewife	3,166 (17%)	62 (24%)
Others (Specify)	210 (1%)	3 (1%)
Regular salaried employment	1,711 (9%)	23 (9%)
Retirement fund/Pension	121 (1%)	1 (0%)
Self-employed	760 (4%)	13 (5%)
Student	6,169 (34%)	90 (34%)
Unemployed/seeking work	690 (4%)	8 (3%)

Out of the 3,350 households interviewed, 1,137 households (34%) have reported that at least one of their members had a fever episode (for more than 7 days) in the last 6 months, and had sought treatment from any external sources. In addition to fever, other symptoms described by respondents had joint pain, retro orbital pain and rashes (29%), loose motion and loss of appetite (27%), joint pain lasting for more than a month (22%) and red spots on the chest (5%). Overall, 512 respondents (15%) declared four of these symptoms and 329 (10%) three of them. Regarding the sources of treatment, more than a third (36%, n = 412) visited a private physician or a clinic, followed by almost an equal proportion (33%, n = 374) who visited a government hospital; about one in every ten cases (13%, n = 143) approached quacks or informal health providers. The main criterion mentioned for the choice is the convenient location of the health facility (46%); other reasons include financial reasons or lower costs of treatment (24%) or habitual choice or past satisfactory experience in seeking treatment from the provider (24%).

**Fig 2:**
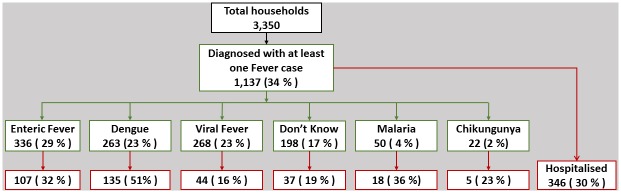
Pathology of respondents diagnosed with fever.

Various pathologies have been diagnosed for the 1,137 respondents (Fig 2). During the 2013 dengue season, 8% of the total respondents or households surveyed had at least one symptomatic and diagnosed dengue case.

In the next section, we will focus on the 263 respondents having one dengue case in their household.

### Sociology of dengue

As per the survey results, 51% (135) of the 263 respondents affected by a dengue case were hospitalised (Fig 2). Among the latter, 47% (64) were diagnosed in one of Delhi’s 39 sentinel hospitals, and then potentially recorded as official dengue cases. Among those hospitalised, the mean number of days of hospitalisation is more for those in governmental hospitals (7.44 days SD ± 8.2) than for private hospitals (5.24 days SD ± 4.9). Dengue treatment expenses were lower in governmental facilities (on an average, a total of 4,691 INR, SD ± 5,655 INR) than in private facilities (on an average, a total of 9,001 INR, SD ± 17,222 INR).

Sex ratio of the dengue cases does not differ significantly from sex ratio of the total population surveyed. This is contrary to several studies which reveal a male to female ratio as high as 3–5:1[25]. One explanation of this gender difference may be found in the bias in healthcare-seeking behaviour, as most of these studies were hospital-based. In the present survey, the ratio of male to female hospitalised persons is close to 1:1.

Age groups that appear to be more at risk include 15–19 year old teenagers and 30–34 year old adults, which is consistent with the patterns observed elsewhere in India [25]. Underrepresentation of dengue cases in small children (3%) compared to their proportion in the population (8%) must be investigated. In fact, as most of them are vulnerable given their lifestyle (they play close to the house amidst the litter and wear hardly any clothes), one could expect that incidence rates in this group would be higher as compared to adult populations. In comparison, a 2013 Delhi study found 22% incidence of new infection only among infants (<1 year) [23].

While social activities are not significantly different among dengue cases and others as the proportion of dengue cases in housewife population (24%) makes a significant contribution (χ^2^ = 5.75) to the value of the computed chi square.

### Knowledge about dengue, mosquito presence in the environment and information sources

Among the 3,350 heads of households, 3,277 (97.8%) have already heard about dengue. These figures are very high and similar to previous studies conducted in Delhi [21–26]. The main source of information of those diagnosed with dengue is word of mouth for 41% of respondents, followed by radio/television (36%) and advertisements/hoardings (13%). 39% of the respondents having only one source of information and 48% gathered information about dengue from two sources. Given that the surveys were not directly related to dengue and that the questionnaire was already long, there were no questions included to qualify the level of this knowledge, in terms of methods of transmission, common symptoms or treatment of the disease. In any case, the objective was mainly focused on linking their perception of mosquito presence in their neighbourhood, the level of fear concerning dengue and their actions to fight against this problem.

A majority of respondents (76%) considers mosquito as a major problem in their living space, such as homes, their vicinity as well as in public places such as parks and markets (74%), but a few report encountering hazards of mosquitoes while commuting by bus, taxi or metro (57%)

This presence of mosquitoes in their environment is linked to a feeling of insecurity as the RSBY household survey confirms that 79% of the households are afraid of dengue spreading to their living spaces such as home, workplace, public places and commuting means. This fear is well understood as it suggests that there is a link between the presence of mosquitoes and the risk of dengue spread. Whether or not this feeling of fear is linked to the environment must be called into question.

The presence of breeding sites indoors or in the neighbourhood increases the probability of emergence of mosquitoes and can, therefore, contribute to this feeling of mosquito abundance. Inside the houses, a major contributor to mosquito production is water storage (jars, water coolers). Outdoors, plants provide resting places for mosquitoes and discarded materials produce breeding habitats. In every surveyed house, the teams noted the presence of discarded materials and vegetation. Only about half the households (50%) have access to piped water supply within their households, while the rest gets water from public taps (21%) and from tube wells or hand pumps (15%). Therefore, a high proportion of the households have to store water inside their homes. For most of the households (71%), average water availability is less than 4 hours per day and more than one in every four households (27%) have to depend on water tankers to meet their water demand for domestic purposes. One in every four (25%) of the 3,350 houses visited, are surrounded by discarded materials, which provides suitable breeding areas for the growth of *Aedes aegypti* [27] and only 5% are surrounded by plants or green spaces.

If we consider only the houses affected by dengue cases, these figures are similar for those surrounded only by plants and gardens/vegetation. But this figure goes up to 36% compared to the total population for the presence of discarded materials such as old plastic products, glass bottles, iron scrap or tyres. In all, 54 households (21%) have both potential mosquito breeding and resting sites in the surroundings.

### Practices followed for prevention of dengue

Despite perception of a strong presence of mosquitoes in their surroundings (76% of the 3,350 households), only 46% declare checking their environment to avoid breeding sites. These figures are similar in the two groups, households with diagnosed dengue cases or those without. Controlling water storage and the removal of garbage are well known practices favouring a safer environment and among the sampled households, more than 36% of houses with dengue cases have discarded materials (old plastic products, glass bottles or iron scrap) inside or in the premises.

To prevent mosquito bites, 27% and 60% use one and two protective measures respectively. Repellents and fans are the most popular measures quoted by the surveyed households, whereas the commonly advocated practice of wearing long sleeves and pants is not at all considered as a means of protection (Table 2). While individual protective measures such as *cream* and *coil* are quoted more by the dengue diagnosed group than the total population, there is no major difference in terms of practices related to prevention of mosquito bites between these two groups.

**Table 2:** Individual protection against mosquito bites in the survey population (N = 3,350) and in dengue household group (N = 263).

Practices against mosquito bites	Total (%)	Dengue group (%)
Coils/Creams/Hit/All-out plugin	78.1	84.4
Wearing long sleeves	6.7	5.3
Using fans	87.2	84.7
Using bed-nets	9.1	7.9
Sleeping covered with sheets	5.1	4.9

## Discussion

The above analysis of the conducted survey reveals that 8% of the surveyed households have reported at least one diagnosed dengue case in 2013. This result can be compared with another study conducted in Delhi where findings show that 10.6% of the people screened had dengue in 2013 [23]. As this later study mixed rich and poor areas as well as symptomatic (37%) and asymptomatic (63%) dengue infections, one can suggest that people living in JJ Clusters were more exposed to dengue in 2013.

However, without an epidemiological study on the proportion of both asymptomatic and symptomatic dengue infections in the JJ settlements population, one cannot strictly compare these figures with the prevalence of dengue population in 2013. In any case, we think that, at least in case of dengue, the health care demand of this population starts with a high disease burden level which explains the fact that most of the dengue cases recorded in this survey are severe hospitalisation cases; this is just the tip of the iceberg of dengue infections in this population. On the basis of these results, it may be assumed that the seroprevalence of dengue must be high in this population and an epidemiological survey should be conducted. This latter could give an epidemiological panorama of this population, including asymptomatic cases and those with mild symptoms as perceived by them and which may act as a reservoir for dengue transmission. Indeed, as mosquito presence is a big issue for JJ clusters, the presence of a single dengue case potentially has a snowball effect among members of the same family and the neighbouring families.

The reason why incidence among small children in this population is so low is not clear. One can make the hypothesis of a bias in the survey. Indeed, none of the respondents have declared more than one dengue case in the household, which suggests that it was the most serious case but do not excludes the fact that other cases have been suspected among the family members but wasn't diagnosed. Epidemiological survey could also throw light on this issue. But another hypothesis of this result observed among the mass residing in the JJ Cluster is that in a context of budget limitation, households operate choices that do not give priority to small children. This behaviour has already been observed when very poor populations have to struggle to survive, when they live precarious lives without decent housing, sanitation or clean water [28].

In comparison with the total population, housewives and young people were more exposed to dengue infection in 2013. One can suppose that this situation is linked to the sedentary life of housewives in a risky environment. Educational programs should focus on this specific population to improve their impact as these women are the ones mostly in charge of water storage and cleanliness of the house and its surroundings.

The perception of mosquito’s abundance and nuisance at home, office and public spaces is very high for 64% to 75% of respondents and should lead to better surveillance and control of breeding sites in these environments. The proportion of houses surrounded by discarded materials is higher in the households with dengue (36%) than in potentially dengue-free houses (24%). This result suggests that even with a dengue experience and potentially an acute perception of the risk and its factors, proper management of environmental conditions is still lacking. There is an urgent need to improve knowledge on the disease and elimination of discarded material to reduce the risk for vector born disease as it has been shown in other places. Thailand reported a significant reduction of DHF in areas where clean-up campaigns were conducted before and during the monsoon [29].

This abundance of mosquitoes goes with an extremely high level of fear of dengue infection among majority of the households, but is not concretized by a high level of preventive practices. The gap between knowledge of dengue and preventive practices (individual measures and breeding control) has already been noted in similar studies carried out in the South Asian region [30–31]. It is also found that less than 50% households declare having used measures to avoid breeding sites of *Aedes* mosquitos although more than 70% of them think that mosquitoes are a major issue in their living space.

The mode of transmission of dengue through *Aedes aegypti* mosquito bites should also be improved. More than 12% of the sampled households responded that they use bed-nets and sleep under bed-sheets to protect themselves against dengue, whereas *Aedes ae*. is a daytime biter. In comparison, less than 6% declare wearing long sleeves and pants, which is nevertheless an effective means of protection against bites. Improvement in the knowledge of this vector and the dengue transmission mode could help to involve the population in a more significant manner.

Among the source of information about dengue, it is found that word of mouth and mass media such TV and radio are by far the main sources of information related to dengue but it does not seem enough to lead to an efficient breeding control and dengue prevention. Even among the households that declared at least one family member infected and hospitalised by dengue fever, these two main factors stays at the first line on the information whereas a health doctor could have been quoted. This should act as a message for health workers’ campaign and municipality workers to give some door-to-door information on how to prevent breeding sites and dengue infection.

Based on this study, we suggest increasing educational programs to improve community awareness about dengue. We also suggest going further in concrete steps with health workers and municipal workers showing how to deal concretely with *Aedes* breeding sites including emptying water containers regularly and applying repellent and protective clothes during the day time are the two basic individual behaviours which can have a major impact at population level. Women must be involved in these campaigns as they are more vulnerable to dengue and they are also responsible for controlling the household environment.

## Supporting information

S1 File Blank copy of the survey used in the study. (PDF)

S2 File Details on the sampling frame. (PDF)

S3 File Data used for analysing relations between environment, knowledge and practices at the level of households. (XLSX)

## References

[1] WHO. Global strategy for dengue prevention and control, 2012–2020. WHO report; 2012.

[2] Bhatt S, Gething P, Brady O, Messina J, Farlow A, Moyes C, et al The global distribution and burden of dengue. Nature; 2013. 10.1038/nature12060 PMC3651993 23563266

[3] Gubler D.J. The economic burden of dengue. Am. J. Trop. Med. Hyg. 2012; 86: 743–4. 10.4269/ajtmh.2012.12-0157 22556068 PMC3335674

[4] Chen LH, Wilson ME. The role of the traveler in emerging infections and magnitude of travel. Med Clin N Am. 2008; 92: 1409–32. 10.1016/j.mcna.2008.07.005 19061759 PMC7094659

[5] Castro M, Sanchez L, Perez D, Sebrango C, Shkedy Z, Van der Stuyft P. The Relationship between Economic Status, Knowledge on Dengue, Risk Perceptions and Practices. PLoS ONE; 2013; 8(12): e81875 10.1371/journal.pone.0081875 24349145 PMC3861357

[6] Gubler DJ. Epidemic dengue/dengue haemorrhagic fever as a public health, social and economic problem in the 21st century. Trends Microbiol. 2002; 10: 100–3. 11827812 10.1016/s0966-842x(01)02288-0

[7] Daudé É., Mazumdar S. Combating Dengue in India: Challenges and Strategies, EPW: Econ Polit Wkly. 2016; 51:8, 77–81.

[8] Balaya S, Paul SD, Lima LV, Pavri KM. Investigations on an outbreak of dengue in Delhi in 1967. Indian J Med Res. 1969; 57: 767–74. 5805380

[9] Diesh P, Pattanayak S, Singha P, Arora DD, Mathur PS, Ghosh TK, et al. An outbreak of dengue fever in Delhi-1970. J Commun Dis. 1972; 4: 13–8.

[10] Rao CVRM, Bagchi SK, Pinto BD, Ilkal MA, Bharadwaj M, Shaikh BH, et al The 1982 epidemic of dengue fever in Delhi. Indian J Med Res. 1985; 82: 271–5. 4077169

[11] Kabra SK, Verma IC, Arora NK, Jain Y, Kalra V. Dengue haemorrhagic fever in children in Delhi. Bull World Health Organ. 1992; 70: 105–8. 1568274 PMC2393341

[12] Dar L, Broor S, Sengupta S, Xess I, Seth P. The first major outbreak of dengue haemorrhagic fever in Delhi, India. Emerg Infect Dis. 1999; 4: 589–90. 10.3201/eid0504.990427 PMC2627747 10458971

[13] Singh NP, Jhamb R, Agarwal SK, Gaiha M, Dewan R, Daga MK, et al The 2003 outbreak of Dengue fever in Delhi, India. Southeast Asian J Trop Med Public Health; 2005; 36: 1174–8. 16438142

[14] Pandey A, Diddi K, Dar L, Bharaj P, Chahar HS, Guleria R, et al. The evolution of dengue over a decade in Delhi, India. J Clin Virol. 2007; 40: 87–8. 10.1016/j.jcv.2007.05.011 17631046

[15] Kumari R, Kumar K, Chauhan LS. First dengue virus detection in Aedes albopictus from Delhi, India: Its breeding ecology and role in dengue transmission. Trop Med Inter Health; 2011; 8: 949–54. 10.1111/j.1365-3156.2011.02789.x 21668590

[16] Afreen N, Deeba F, Naqvi I, Shareef M, Ahmed A, Broor S, et al Investigation of 2013 Dengue Fever Outbreak from Delhi, India. PLOS Currents Outbreaks; 2014. 10.1371/currents.outbreaks.0411252a8b82aa933f6540abb54a855f PMC4169351 25642359

[17] National Vector Borne Disease Control Program. http://nvbdcp.gov.in/den-cd.html

[18] Telle O, Vaguet A, Yadav N K, Lefebvre B, Daudé E, Paul R., et al The spread of dengue in an endemic urban milieu—the case of Delhi, India. PLoS ONE; 2016; 11(1): e0146539 10.1371/journal.pone.0146539 26808518 PMC4726601

[19] Acharya A, Goswami K, Srinath S, Goswami A. Awareness about dengue syndrome and related preventive practices amongst residents of an urban resettlement colony of south Delhi. Journal of Vector Born Diseases; 2005; 42(3): 122–7. 16294811

[20] Singh R, Mittal P, Yadav N, Gehlot O, Dhiman R. Aedes aegypti indices and KAP study in Sangam Vihar, south Delhi, during the XIX Commonwealth Games, New Delhi, 2010. Dengue Bull. 2011; 35: 131–40.

[21] Chinnakali P, Gurnani N, Upadhyay RP, Parmar K, Suri TM, Yadav K. High Level of Awareness but Poor Practices Regarding Dengue Fever Control: A Cross-sectional Study from North India. N Am J Med Sci. 2012; 4(6): 278–82, 10.4103/1947-2714.97210 22754880 PMC3385365

[22] Spiegel J, Bennett S, Hattersley L, Hayden M, Kittayapong P, Nalim S et al Barriers and Bridges to Prevention and Control of Dengue: The Need for a Social–Ecological Approach. EcoHealth. 2005; 2: 273.

[23] Vikram K, Nagpal BN, Pande V, Srivastava A, Saxena R, Anvikar A, et al An epidemiological study of dengue in Delhi, India. Acta Trop. 2015; 153: 21–27. 10.1016/j.actatropica.2015.09.025 26433076

[24] Mazumdar S, Rustagi P, Kumar A. Equity impacts of a targeted health insurance scheme: New evidence from India’s Rashtriya Swasthya Bima Yojana. Report of Institute of Human Development; 2014.

[25] Chakravarti A, Arora R, Luxemburger C. Fifty years of dengue in India. Trans R Soc Trop Med Hyg. 2012; 106: 273–82. 10.1016/j.trstmh.2011.12.007 22357401

[26] Acharya A, Goswami K, Srinath S, Goswami A. Awareness about dengue syndrome and related preventive practices amongst residents of an urban resettlement colony of south Delhi. Journal of Vector Born Diseases; 2005; 42: 122–7. 16294811

[27] Vikram K, Nagpal B, Pande V, Srivastava A, Gupta S, Anushrita, et al Comparison of Ae. aegypti breeding in localities of different socio-economic groups of Delhi, India. Int J Mosq Res. 2015; 2(3): 83–8.

[28] Scheper-Hughes N. Death Without Weeping: The Violence of Everyday Life in Brazil. University of California Press; 1993.

[29] Van Benthem BH, Khantikul N, Panart K, Kessels PJ, Somboon P, Oskam L. Knowledge and use of prevention measures related to dengue in northern Thailand. Trop Med Int Health; 2002; 7: 993–1000. 12390606 10.1046/j.1365-3156.2002.00950.x

[30] Hairi F, Ong CH, Suhaimi A, Tsung TW, bin Anis Ahmad MA, Sundaraj C, et al A Knowledge, attitude and practices (KAP) study on dengue among selected rural communities in the Kuala Kangsar district. Asia Pac J Public Health; 2003; 15: 37–43. 14620496 10.1177/101053950301500107

[31] Itrat A, Khan A, Javaid S, Kamal M, Khan H, Javed S, et al Knowledge, Awareness and Practices Regarding Dengue Fever among the Adult Population of Dengue Hit Cosmopolitan. PLoS ONE; 2008; 3(7): e2620 10.1371/journal.pone.0002620 18612437 PMC2440812

