# Supplementary material for: Widespread fear of dengue transmission but poor practices of dengue prevention: A study in the slums of Delhi, India
Source: PLoS One. 2017 Feb 10;12(2):e0171543. doi: 10.1371/journal.pone.0171543 (PMC5302449; doi:10.1371/journal.pone.0171543)
Supplement: S1 File — (PDF) [file pone.0171543.s001.pdf]

HEALTH INSURANCE IMPACT EVALUATION SURVEY (HIIES)
MAIN QUESTIONNAIRE – BENEFICIARY (SET – C)

Sponsored by

Indian Council of Medical Research (ICMR),
Ministry of Health & Family Welfare, Government of India

INSTITUTE FOR HUMAN DEVELOPMENT
NIDM Building, 3rd Floor, IIPA Campus, I.P Estate, Mahatma Gandhi Marg, New Delhi-110 002, Phones – 2335 8166, 2332 1610 / Fax : 23765410
Email: mail@ihdindia.org

Date: ……/………/………/
दिनांक : ………/………/………/

Investigator Name: ……………………………
शोध कर्मी का नाम :……………………………………………

Stratum & Household Identification
स्तर एवं घर की पहचान

Stratum Identifier Code<br>स्तर पहचानकर्त्ता का कोड	N1<br>(1)	N2a<br>(2)	N2b<br>(3)	N2c<br>(4)	N3a<br>(5)	N3b<br>(6)	N3c<br>(7)	N4a<br>(8)	N4b<br>(9)	N4c<br>(10)	N5a<br>(11)	N5b<br>(12)	N5c<br>(13)
Household serial number<br>मकान क्रम संख्या													

Informed Consent Statement

***INTERVIEWER** – (Read out): Namaskar. My name is (please say your name here) and I work for Institute for Human Development, a research institute in New Delhi. We had earlier visited your household to collect some information regarding your health condition, socioeconomic status and possession of Government health insurance cards/schemes. Now, your household has been selected to collect some further detailed information on health and health care aspects and the amount you have spent on health care for your family members. It will take about 45 minutes for you to answer the questions. You can choose not to answer any questions or refuse participating in the interview at any point of time throughout the interview. The information you provide will be confidential and used for research purposes only. If you can spare the time now, and with your permission, can I start the interview now?*

Oral consent give (Circle one):<br>मौखिक सहमति दी गई (एक को घेरें) :	Yes – *Continue*<br>हाँ– प्रारंभ करें	No -- *Stop the survey*<br>नहीं– सर्वे बंद कर दें	
**Visit Details**	1st Visit	Repeat Visit	Replace/Change

SECTION 1: HOUSEHOLD MEMBER ROSTER

खंड 1 : मकान के सदस्यों का रोस्टर

A. Demographics and Socioeconomics

क. जनसांख्यिकीय एवं सामाजिक अर्थशास्त्र

Details of all family members: In order to determine whom to interview, I need to know who lives at this address. Let me assure you that any information you provide is strictly confidential. I would like the age, sex, education, marital status and relationship to you of each of the members of this household who live here. Please include people who may presently be in an institution due to their health (hospital, nursing home etc.) for a short or long period. All the members in the household should be entered first, from oldest to youngest.Don't forget to include yourself in the appropriate order.

परिवार के सभी सदस्यों के बारे में सूचना : यह तय करने के लिए कि किसका साक्षात्कार करना है, मुझे यह जानने की जरूरत है कि इस पते पर कौन रहता है। मैं आपको आश्वस्त करना चाहता हूँ कि आपके द्वारा दी गई सूचनाएं पूरी तरह गोपनीय होंगी। मैं इस मकान में रहने वाले प्रत्येक सदस्य की उम्र, लिंग,शिक्षा, विवाह की स्थिति एवं आपके साथ उनके संबंध के बारे में जानना चाहूँगा। कृपया उन लोगों को भी शामिल करें जो वर्त्तमान में अपने स्वास्थ्य कारणों से अस्पताल, नर्सिंग होम आदि संस्था में थोड़े या लंबे समय से भर्ती हैं। सबसे पहले मकान में रहने वाले सबसे बुजुर्ग स`चल कर सबसे कनिष्ठ, से संबंधित सूचनाएं अंकित की जाएं।

**1.1** Line No.	**1.2** Name नाम	**1.3** Sex लिंग	**1.4** Relationship with household head मकान के मुखिया के साथ संबंध	**1.5** Age उम्र	**1.6** Marital status विवाह की स्थिति	**1.7** Highest educational level उच्चतम शैक्षणिक स्तर	**1.8** Usual activity status सामान्य किया स्थिति	**1.9** Whether suffered from any minor illnesses during **last 1 month** क्या विगत **एक महीने में** किसी छोटी बीमारी से पीड़ित हुए	**1.10** Whether suffers from any chronic diseases since **last 12 months**? क्या **पिछले 12महीने** में किसी असाध्य रोग से ग्रस्त है ?	**1.11** Whether hospitalized ever during **last 12 months** क्या विगत 12 **महीनों** में अस्पताल में भर्ती होना पड़ा?	**1.12** Whether member smokes regularly (only adult 15+ क्या परिवार के सदस्य नियमित धूम्रपान करते हैं **(केवल 15 वर्ष के उपर के व्यस्क)** (सप्ताह में कम से कम एक या दो बार)	**1.13** Whether member consumes any other form of tobacco regularly क्या सदस्य किसी अन्य प्रकार के तंबाकू का भी सेवन करते हैं? (सप्ताह में कम से कम एक या दो बार)	**1.14** Whether member drinks alcohol (at least once/twice a week) क्या सदस्य शराब पीते हैं (सप्ताह में कम से कम एक या दो बार)	**1.15** Any health Insurance
1														
2														
3														
4														
5														
6														
7														
8														
9														

CODES

For 1.3: ***Sex लिंग :*** *male=1, female=2*

For 1.4: Relation to head **परिवार के मुखिया से संबंध:** *self=1, spouse of head=2, married child=3, spouse of married child=4, unmarried child=5, grand child=6, father/mother/father-in-law/mother-in-law=7, brother/sister/brother-in-law/sister-in-law/other relative=8, servant/employees/other non-relatives=9*

For 1.6: Marital status **विवाह की स्थिति** *: Unmarried=1, Married=2, widowed=3, divorced/separated=4*

For 1.7: Educational level **शैक्षणिक स्तर:** *Illiterate=01; literate but never gone to school=02, Upto class Fifth=03, Upto class Eight=04, Completed class Tenth=05, completed class 12= 06, completed Graduation= 07, Any higher education above graduation(PG)=08*

For 1.8: Usual activity status **प्रतिदिन कार्य स्थिति:** *Casual/daily wage laborer=01, Self-employed (including small business & trade)=02, Regular salaried employment=03, Unemployed/seeking work=04, Student=05, Home-maker/household work/domestic duties (unpaid)=06, Disabled/too Old/too Young=07, Household entrepreneur (tailoring/weaning/hand wash)=08, Hosewife=9, retirement fund/Pension=10, Others=99 (Specify)*

For Q.1.9 to 1.15*: Yes=1, No=0, don't know/can't say=99.*

Note:

Q1.9: *When suffered from any common/minor/short-term illness in last 30 days* ***(such as – fever/cold & cough/loose motion…….etc.)***

विगत 30 दिनों के अंदर किसी सामान्य/छोटी/अल्पकालिक रोग से पीड़ित हुए **(जैसे सर्दी एवं खाँसी ……… आदि)**

Q 1.10: Diabetes/cancer/hypertension/heart alignments/heart alignment/respiratory problems/tuberculosis/arthritis/long standing pain in bone/joints)

क्या **मधुमेह/कैंसर/उच्च रक्तचाप/हृदय रोग/हृदय से संबंधित रोग/श्वास की तकलीफें/तपेदिक/गठिया/जोड़ों का दर्द** लंबे समय से चला आ रहा है?

SECTION 2: HEALTH MEASUREMENT (EQ-5D-3L) [If Required Proxy Respondents – Note Line Number Below]

Respondent Line No.	

नीचे के हर समूह में एक बौक्स में सही का निशान लगा कर, कृपया ये अन्दाज़ा दीजिये कि कौन से कथन आज आप की सेहत के हाल का सबसे अच्छा बयान करते हैं। **[Put ✓ Mark]**

1	**चलना फिरना**	
	मुझे चलने फिरने में कोई मुश्किल नहीं है।	
	मुझे चलने फिरने में कुछ मुश्किल है।	
	मैं बस बिस्तर पर पड़ा/पड़ी हूं।	
2	**अपनी देखभाल**	
	अपनी देखभाल करने में मुझे कोई मुश्किल नहीं है।	
	मुझे अपने आप नहाने धोने या कपड़े बदलने में कुछ मुश्किल है।	
	मैं अपने आप नहाने धोने या कपड़े बदलने में असमर्थ हूं।	
3	**आपकी रोज़मर्रा की गतिविधियां:**<br><br>(**जैसे काम, पढ़ाई लिखाई, घर के कामकाज, पारिवारिक या फुरसत की गतिविधियां**)	
	अपनी रोज़मर्रा की गतिविधियां करने में मुझे कोई मुश्किल नहीं है।	
	अपनी रोज़मर्रा की गतिविधियां करने में मुझे थोड़ी मुश्किल है।	
	मैं अपनी रोज़मर्रा की गतिविधियां करने में असमर्थ हूं।	
4	**दर्द/तकलीफ**	
	मुझे कोई दर्द या तकलीफ नहीं है।	
	मुझे कुछ दर्द या तकलीफ है।	
	मुझे बेहद दर्द या तकलीफ है।	
5	**घबराहट/उदासी**	
	मैं घबराया हुआ या उदास नहीं हूं।	
	मैं थोड़ा घबराया हुआ या उदास हूं।	
	मैं बेहद घबराया हुआ या उदास हूं।	

लोगों को इस बात के बयान करने में मदद के लिये कि उनकी स्वास्थ्य-स्थिति कितनी अच्छी या ख़राब है, हमने एक पैमाना बनाया है (किसी थर्मामीटर की तरह) जिस पर सब से अच्छी कल्पित स्वास्थ्य-स्थिति का आंकन है 100 और सब से ख़राब कल्पित स्वास्थ्य-स्थिति का आंकन 0 है।

हम चाहते हैं कि इस पैमाने पर, आप हमें इस बात का अन्दाज़ा दें कि आप की राय में आज आप की सेहत कितनी अच्छी या ख़राब है। कृपया आप ऐसा नीचे बौक्स से पैमाने के उस बिन्दु तक लाइन खींच कर करें जो ये दर्शाये कि आज आप की सेहत कितनी अच्छी या बुरी है।

आज आप की
स्वास्थ्य- स्थिति

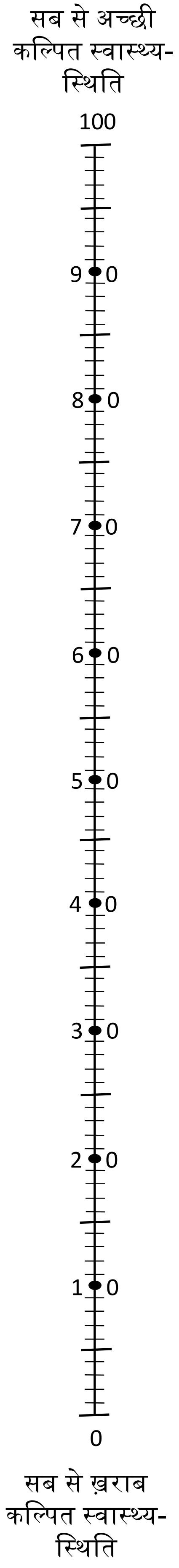

Health-related Quality of Life (HRQOL) [ONLY FOR RESPONDENTS/ NO PROXY RESPONSE]

स्वास्थ्य संबंधी जीवन की गुणवत्ता (एच.आर.क्यू.ओ.एल.)

1	How satisfied are you with your health, **during the last 4 weeks/1 month**?<br>विगत 4 सप्ताह में आप अपने स्वास्थ्य से कितना संतुष्ट हैं ?	1<br>Very dissatisfied<br>बहुत असंतुष्ट	2<br>असंतुष्ट	3<br>Moderate<br>औसत	4<br>संतुष्ट	5<br>Very Satisfied<br>बहुत संतुष्ट
2	How much is your need for medical treatment/help to function in your daily life?<br>आपके दैनिक जीवन में चिकित्सकीय उपचार/मदद की कितनी जरूरत है ?	1<br>Very much<br>बहुत अधिक जरूरत	2<br>जरूरत है	3<br>Moderate<br>औसत	4<br>A Little<br>थोड़ा	5<br>Not at all<br>बिलकुल नहीं
3	How safe do you feel in your daily life?<br>आप अपने दैनिक जीवन कितना सुरक्षित महसूस करते हैं ?	1<br>Not at all<br>बिलकुल नहीं	2<br>A Little<br>थोड़ा	3<br>Moderate<br>औसत	4<br>सूरक्षित	5<br>Very safe<br>बहुत सूरक्षित
4	Do you generally have enough money to meet your daily/regular needs?<br>क्या आमतौर पर आपको अपने दैनिक जीवन की जरूरतों को पूरा करने के लिए प्रर्याप्त पैसे होते हैं ?	1<br>Not at all<br>बिलकुल नहीं	2<br>A Little<br>थोड़ा	3<br>Moderate<br>औसत	4<br>Mostly<br>अधिकांश	5<br>Completely<br>पूरी तरह
5	How important is religion/spirituality to you in your daily life, in connection with your normal daily activities?<br>आपकी दिनचर्या में धर्म/अध्यात्म का कितना महत्व है ?	1<br>Not at all<br>बिलकुल नहीं	2<br>A Little<br>थोड़ा	3<br>Moderate<br>औसत	4<br>महत्वपूर्ण	5<br>Extremely Important<br>अति महत्वपूर्ण
6	How satisfied are you with your son(s)<br>आप अपने बेटों से कितना संतुष्ट हैं ?	Very dissatisfied<br>बहुत असंतुष्ट<br>1	2<br>असंतुष्ट	3<br>Moderate<br>औसत	4<br>संतुष्ट	5<br>Very Satisfied<br>बहुत संतुष्ट
7	How satisfied are you with your daughter(s)<br>आप अपने बेटियों से कितना संतुष्ट हैं ?	1<br>Very dissatisfied<br>बहुत असंतुष्ट	2<br>असंतुष्ट	3<br>Moderate<br>औसत	4<br>संतुष्ट	5<br>Very Satisfied<br>बहुत संतुष्ट

8	How satisfied are you with the following aspects of your daily life:<br>*(1=Very dissatisfied, 2=Somewhat dissatisfied, 3=Neither, 4=Somewhat satisfied, 5=Very satisfied)*<br>आप अपने दैनिक जीवन के निम्नलिखित पहलुओं से कितना संतुष्ट हैं :<br>**(1 = बहुत असंतुष्ट ,2= कुछ हद तक असंतुष्ट, 3= कोई नहीं, 4= कुछ हद तक संतुष्ट, 5= बहुत संतुष्ट)**					
	A. Activities you do for your daily living (schooling/hh work domestic work included)<br>आपके दैनिक जीवन की गतिविधियाँ (स्कूल संबंधी/एच.एच. कार्य, घरेलू कार्य शामिल)	**1**	**2**	**3**	**4**	**5**
	B. Your capacity for work आपके कार्य करने की क्षमता	**1**	**2**	**3**	**4**	**5**
	C. Your capacity to bear sudden major expenses औचक खर्चे को पूरा करने की आपकी क्षमता	**1**	**2**	**3**	**4**	**5**
	D. Support/help you get from friends/neighbours/colleagues<br>आपको अपने मित्रों/पड़ोसियों/सहकर्मीयों से प्राप्त समर्थन/मदद	**1**	**2**	**3**	**4**	**5**
	E. Conditions of your living place/surroundings आपके निवास स्थल/परिवेश की स्थिति	**1**	**2**	**3**	**4**	**5**
	F. Access to health facilities स्वास्थ्य सुविधाओं तक पहुँच	**1**	**2**	**3**	**4**	**5**
	G. Transport facilities you mostly use परिवहन सुविधाएं जो आप अधिकांशतः प्रयोग करते हैं	**1**	**2**	**3**	**4**	**5**
	H. Safety/security of your locality/community आपके मुहल्ले/समुदाय की देखरेख/सुरक्षा	**1**	**2**	**3**	**4**	**5**

Social Networks सामाजिक नेटवर्क

	Is anyone in the family presently a member of any of the followings :<br>क्या परिवार का कोई भी एक सदस्य निम्नलिखित में से किसी का भी सदस्य है :			
9	Self Help Group (SHG)/Credit Cooperatives स्वंय सहायता समूह (एस.एच.जी.)/कर्ज देने वाली सहकारी संस्थाएँ	**Yes (1)**	**No (0)**	**DK/CS (99)**
10	NGO/MFI (Micro Finance Institution) client एन.जी.ओ./एम.एफ.आई.(लघु वित्त संस्थान) ग्राहक	**Yes (1)**	**No (0)**	**DK/CS (99)**
11	Trade unions मजदूर संगठन	**Yes (1)**	**No (0)**	**DK/CS (99)**
12	Political party राजनीतिक दल	**Yes (1)**	**No (0)**	**DK/CS (99)**
13	Religious organization धार्मिक संगठन	**Yes (1)**	**No (0)**	**DK/CS (99)**
14	Local CBO's (Community Based Organizations) स्थानीय सी.बी.ओ.(समुदाय आधारित संगठन)	**Yes (1)**	**No (0)**	**DK/CS (99)**
15	Other membership-based organization सदस्यता आधारित अन्य संगठन	**Yes (1)**	**No (0)**	**DK/CS (99)**
16	Do you or your family members personally know the Nigam Parishad/MLA or any ward member of your AREA? क्या आप/आपके परिवार के सदस्य निगम पार्षद/एम.एल.ए.या अपने क्षेत्र के किसी वार्ड सदस्य को व्यक्तिगत रूप से जानते हैं ?	**Yes (1)**	**No (0)**	**DK/CS (99)**
	Number of times you did the following during last 7 days/in last week?<br>विगत **7 दिनों/एक सप्ताह** के अंदर निम्नांकित में से कौन सा आपने कितनी बार किया ?			
17	Watch TV टेलीविजन देखना	**Never (1)**	**Rarely (2)**	**Daily (3)**
18	Listen Radio रेडीयो सुनना	**Never (1)**	**Rarely (2)**	**Daily (3)**
19	Read Newspaper/magazines समाचार पत्र/पत्रिकाएं पढ़ना	**Never (1)**	**Rarely (2)**	**Daily (3)**
20	What are your main sources of information regarding government programmes/schemes?<br>सरकारी कार्यक्रमों/योजनाओं के बारे में सूचनाओं का आपका मुख्य स्रोत क्या है ?<br>**(*Multiple responses possible)***<br>*[Friends and family-members मित्रों एवं परिवार के सदस्यों=1,*<br>*Media (A-V/Print मीडिया(श्रव्य–दृश्य/अखबार)=2,*<br>*Local government workers (ANM, AWW स्थानीय सरकार के कर्मी (ए.एन.एम.,ए.डब्ल्यू. डब्ल्यू.))=3,*<br>*Political party, workers/leaders/elected reps राजनीतिक दल,कर्मी/नेता/निर्वाचित प्रतिनिधि=4,*<br>*Religious leaders/places of worship धार्मिक नेता/पूजा स्थल=5,*<br>*Local NGO/CBO members स्थानीय/एन.जी.ओ./सी.बी.ओ. सदस्य=6,*<br>*Others(specify) अन्य(स्पष्ट करें)=98, Don't Know/CS नहीं जानते=99*			

SECTION 3 HEALTH STATUS AND HEALTH CARE UTILIZATION स्वास्थ्य की स्थिति एवं स्वास्थ्य के देखभाल का लेखा–जोखा

A. Fevers बुखार [ONLY FOR RESPONDENTS/ NO PROXY RESPONSE]

21	During the last 6 months (after last Diwali), have you/your child had any fever which lasted for more than 7 days?<br>पिछली दीपावली के बाद विगत 6 महीने के अंदर क्या आपको या आपका बच्चा को कभी ऐसा बुखार हुआ जो 7 दिनों से ज्यादा रहा हो ?	Yes (1)	No (0)	If No Skip to next Sub-Section यदि नही तो अगले भाग में जाएं
22	Line number of ailing child/self बीमार बच्चे/स्वंय की लाइन संख्या			
23	Please indicate the number of times you had such episodes of fever (>7days)<br>इस जैसे बुखार के दौरे (7 दिनों से अधिक) को कृप्या संख्या में इंगित करें।			
	Please indicate whether you had experienced the following symptoms/conditions during the fever episode(s)<br>बार बार बुखार आने के दौरान क्या आपने निम्नांकित लक्षणों/स्थितियों का अनुभव किया, कुपया बताएं (*Probe – read out*) पढकर पूछें			
24	Loose motions, loss of appetite<br>पतला दस्त, भूख खत्म होना	Yes (1)	No (0)	DK/CS (99)
25	Red spots on the chest<br>छाती पर लाल धब्बे	Yes (1)	No (0)	DK/CS (99)
26	High temperature, Pain in the joints, headache (behind the eyes), Rashes<br>तेज ज्वर, जोड़ो में दर्द, सिरदर्द (आँखों के पीछे), दाने निकल आना	Yes (1)	No (0)	DK/CS (99)
27	Pain in the joints lasting for more than a month<br>एक महीने से खींच रहा जोड़ों में दर्द	Yes (1)	No (0)	DK/CS (99)
28	Did you consult any physicians/hospitals/clinics for treatment?<br>क्या आपने उपचार के लिए किसी चिकित्सक/अस्पताल/क्लीनिक से परामर्श लिया ?	Yes (1)	No (0)	If No skip to next Sub-Section यदि नही तो अगले भाग में जाएं

29	Type of provider प्रदाता का प्रकार	Govt. clinic/dispensary सरकारी क्लिनीक/डिसपेंसरी =01
		Govt hospital सरकारी अस्पताल=02
		Private physician/clinic निजी चिकित्सक/क्लिनीक =03
		Private hospital/nursing home (including NGO/trust hospitals) निजी अस्पताल/नर्सिंग होम(एन.जी.ओ./ट्रस्ट अस्पतालों से सहित) =04
		Quacks/informal providers फर्जी चिकित्सक/असंगठित प्रदाता =05
		Medicine shops/pharmacy/old prescriptions दवाखाना/फार्मेसी/पूराने नुस्खे =06
		Hakim/vaids हकीम/वैद्य =07
		Other (specify) अन्य (स्पष्ट करें) =98
30	Name of the facility/doctor visited<br>सुविधा का नाम/ चिकित्सक जिसने देखा है<br>(Note: 'Name' during interview and Fill-up type from local mapping)<br>चिकित्सक का नाम साक्षात्कार के दौरान लिखें एवं प्रकार स्थानीय स्तर पर पता लगाकर भरें	
31	Reasons for seeking treatment from the provider/facility mentioned<br>प्रवंधक से उपचार/चिह्नित सुविधा प्राप्त करने का कारण	Nearest health facility/convenient (including convenient operational hours) निकटतम स्वास्थ्य सुविधा/सूविधाजनक (संचालन के सुविधाजनक घंटे) =01
		Low cost/financial reasons कम लागत/वित्त =02
		Usual choice for treatment/past experience of effective treatment उपचार का नियमित विकल्प/अतीत के प्रभावी उपचार का अनुभव =03
		neighbours/friends advised पड़ोसियों/मित्रों का सलाह =04
		Others (specify) अन्य(स्पष्ट करें) =98
		Not sure/DK/CS) =99

32	Did you get any blood tests done? क्या आपने किसी भी प्रकार का खून जाँच करवाया ?	Yes (1)	No (0)

33	Were you informed/did you know the results of the tests?<br>क्या आपको जाँच का परिणाम सूचित किया गया था/परिणाम पता था ?	**Yes (1)**	**No (0)**
33A	What was the diagnosis?<br><br>(dengue-1, chickngunya-2, malaria-3, enteric fever/typhoid-4, viral fevers-5, DK/CS-99)		
34	Are you aware of what medicines you were given/prescribed?<br>क्या आप जानते थे कि आपको क्या दवाएं दी गईं<br><br>*If available, check the prescription and note.*<br>यदि नुस्खे का कगज उपलब्ध हो तो उसकी जाँच कर एवं नोट कर लें।	**Yes (1)**	**No (0)**
35	Were you required to be hospitalized?<br>क्या आपको अस्पताल में भर्ती करने की जरूरत थी ?	**Yes (1)**	**No (0)**
36	Number of days hospitalized?<br>कितने दिनों तक अस्पताल में भर्ती रहे ?		
37	Type of hospital where admitted (Name and Type of hospital/Clinic)<br><br>अस्पताल का प्रकार जहाँ भर्ती हुए (अस्पताल का नाम एवं प्रकार)		

Now, I would like to collect some information on the expenses incurred for your treatment for Fevers
From the illness you have been describing, I will require expenses incurred on different purposes, and also from where/which sources did you manage to pay. **(In case of multiple episodes, information from the most recent)**

अब मै आपुसे आपके बुखार के) उपचार पर हुए खर्च/जिस बीमारी के बारे में आप विवरण देते रहें हैं से संबंधित कुछ सूचनाएं संकलित करना चाहूँगा|
(कई बार बीमार होने की स्थिति में सबसें ताजा दौर से संबंधित सूचनाएं)

	Please provide amount spent for<br>कृपया खर्च की गए राशि के बारे में बताएं<br>**(For Fevers बुखार)**<br>**Col (1)**	Total cost<br>कुल लागत<br>**Col (2)**	Amount paid out-of pocket (own income/savings)<br>दी गई राशि (अपनी आय/बचत)<br>**Col (3)**	Amount from other sources<br>अन्य स्रोतों से राशि<br>**Col (4)**	Source<br>स्रोत<br>**Col (5)**	Amount provided under RSBY scheme आर.एस.बी. वाई.योजना के तहत दी गई राशि<br>**Col (6)**
38	Doctor's fee<br>चिकित्सक का शुल्क					
39	Diagnostic tests<br>निदान के लिए जाँच					
40	Medicine/injections<br>दवाई/सूईयाँ					
41	Bed charges<br>बेड शूल्क					
42	Surgery charges<br>शल्य चिकित्सा शुल्क					
43	Any special diet/other food<br>कोई विशेष आहार/अन्य भोजन					
44	Follow-up costs<br>आगे की जाँच की लागत					
45	Transportation (including ambulance)<br>परिवहन (एम्बुलेंश सहित)					
46	Any other indirect costs (wage loss of attendants etc.)<br>कोई और अप्रत्यक्ष खर्च (सेवक को पारिश्रमिक का नुकसान आदि)					
47	**Total expenses**<br>कुल खर्च					

Instructions: निर्देश

For 3: Ask respondent whether entire amount was met with self-income/salary/from other household member. If so, copy the total costs from Col.II to Col. III and skip col.IV & col.V

उत्तरदाता से पूछें कि क्या पूरी राशि खुद की आय/वेतन/घर के अन्य सदस्य से पूरी की गई। यदि हाँ, कॉलम 4 एवं कॉलम 5 को छोड़ कॉलम 2 से कॉलम 3 में लिखित कुल खर्चे को नकल कर लें।

For 4: Entre the amount that was with supported from other sources:

अन्य स्रोतों से प्राप्त कुल राशि को प्रविष्ट कर लें :

If Partial support=include amount यदि आंशिक मदद=राशि शामिल करें

If total (entire amount supported by external sources)=copy Col. 2.

यदि योग (वाह्य स्रोत से प्राप्त कुल राशि)=कालम 2 की नकल कर लें

For 5: Other/External sources: अन्य/बाह्य स्रोत :

1=Borrow from friend/neighbor/relatives; मित्रों/पड़ोसियों/रिश्तेदारों से कर्ज

2=Loans from office; दफतर से उधार

3=Paid by the employee कर्मचारी द्वारा भुगतान

4=Loan (with interest) from money lender साहूकार से लिया गया सूद के साथ उधार

9=Others (specific) अन्य (स्पष्ट करें)

48	How were the expenses met? (*Multiple responses possible)*<br><br>खर्चे कैसे पूरे किये गए ? (अनेक उत्तर संभव हैं)<br><br>*Note: These questions are to be asked if the respondent is unable to give break-up of the expenses incurred (and its financing sources)* नोट : ये प्रश्न तब पूछे जाने है *यदि उत्तरदाता खर्च (उसके वितीय स्रोत)टुकड़ों में नहीं बता पता है*	*Self savings* खुद की बचत *=1*<br>*Employer paid* नियोक्ता द्वारा देय *=2*<br>*Friends/family* मित्रों/रिश्तेदारों *=3*<br>*Health ins-RSBY* स्वास्थ्य में आर.एस.बी.वाई. *=4*<br>*Health insurance* स्वास्थ्य बीमा *(others* अन्य*)=5*<br>*Borrowings from money lenders with interest* साहूकारों से सूद सहित लिया गया कर्ज *=6*<br>*Selling of assets* संपत्ति बेचकर *=7*<br>*Others* (specify) अन्य *=98*<br>*DK/CS=99*
49	Was it of any difficulty for your family/yourself to meet the costs associated with your (child's) treatment, and spending the amount you mentioned?<br><br>क्या आपके/आपके परिवार के लिए आपके बच्चे के उपचार पर खर्च, जिसके बारे में आपने उल्लेख किया, करने में किसी तरह की कठिनाई हुई ?	*1=Extremely difficult* अत्याधिक कठिनाई<br>*2=Somewhat difficult* कुछ हद तक कठिनाई<br>*3=Not at all* बिलकुल नहीं
50	Number of days confined to bed/absent in work<br><br>आप कितने दिनों तक काम पर नहीं जा सके/बिस्तर पर पड़े रहे ?	

B. Dengue /chikungunya

Note: ASK TO ALL RESPONDENTS (सभी उत्तरदाताओं से पूछें)

51A	Have you heard about Dengue?	Yes (1)	No (0)
51B	If yes, what were the sources from where you heard about Dengue? (*Multiple responses possible)*	Radio/TV	1
		Newspapers	2
		Advertisement/Hoardings	3
		Religious Place	4
		Political parties	5
		Word of mouth	6
		Others(Specify) ______	98

51C	How would you describe the problems of mosquitoes in the following places: Would you say it is: **CODE:** Extreme-1, Somewhat high-2, Higher at times/occasionally-3, Low-4, Negligible/absent-5	
		I. At your home
		II. At your place of work
		III. In transport/while commuting
		IV. In public places (parks, markets)

51D	Do you use any mosquito repellants/preventions?		
	If so, what types of repellants/methods **[Put ✓ Mark]** (***Multiple responses possible)***	1	None
		2	Sleep being covered under sheets
		3	Use of Bed-nets
		4	Using Fans
		5	Coils/Creams/Hit/All-out plugin
		6	Wear long sleeves
		98	Others (Specify) ______

51F	Where do you sleep at night?	Closed rooms – (1)
		Court-yards – (2)
		In open-spaces – (3)
		98-Others ______

51G	क्या आपको पता है कि डेंगू/चिकनगुनिया की रोकथाम कैसे की जाती है ? (***Multiple responses possible)*** **अगर हाँ–तो कृपया दो तीन उपाय बताएं**	Yes (1)	No (0)	1.
				2.
				3.

51H	क्या आप जहाँ रहते हैं उस स्थान/जगह के वातावरण/माहौल में आप डेंगू/चिकनगुनिया होने का खतरा महसूस होता है?	Yes (1)	No (0)	DK/CS(99)
51I	Do you regularly check your house to control mosquito breeding sites?	Yes (1)	No (0)	

**OBSERVE AND NOTE – DO NOT ASK**			
51J	Are there any plants or gardens/vegetation around the house?	Yes (1)	No (0)
51K	Are there any discarded materials (old plastic/glass bottle/iron scrap/tyres)?	Yes (1)	No (0)

C. Respiratory Ailments/Problems श्वास संबंधी रोग/समस्याएं [ONLY FOR RESPONDENTS/ NO PROXY RESPONSE]

53	During the last 12 months (after last Holi), have you/ your child experienced any problem/difficulty with your breathing?<br><br>पिछली होली के बाद के विगत 12 महीनों में क्या आपने या आपका बच्चा साँस लेने में किसी समस्या/कठिनाई का अनुभव किया है ?	**Yes (1)**	**No (0)**	**DK/CS (99)**
54	Line number of ailing child/self<br><br>बीमार बच्चे/स्वंय की लाइन संख्या			
During the last 12 months, have you/ your child experienced any of the following symptoms:<br><br>पिछले 12 महीनों में निम्नांकित में से कोई लक्षण आपने या आपका बच्चा ने अनुभव किया है ?				
55	Attacks of wheezing or whistling sounds while breathing<br>श्वास लेने के दौरान घरघराहट या सीटी की सी आवाज का प्रकोप	**Yes (1)**	**No (0)**	**DK/CS (99)**
56	Wheezing, which comes after any physical activity/exercise<br><br>किसी भी शारीरिक गतिविधि/अभ्यास के बाद घरघराहट का होना	**Yes (1)**	**No (0)**	**DK/CS (99)**
57	Any feeling of tightness in your chest<br><br>आपकी छाती में किसी प्रकार के जकड़न का अनुभव	**Yes (1)**	**No (0)**	**DK/CS (99)**
58	Waking up in the morning/any time with a feeling of tightness in chest<br><br>प्रातःकाल/किसी समय नींद से उठने के साथ छाती में जकड़न के अनुभव का होना	**Yes (1)**	**No (0)**	**DK/CS (99)**
59	Attack of shortness of breath that comes without any physical activity<br><br>बिना किसी शारीरिक श्रम के तेजी से श्वास चलना	**Yes (1)**	**No (0)**	**DK/CS (99)**
For above Questions Q 53, Q 055-Q 059 if answers is '0'or '99', SKIP to Sub-Section 'D';<br><br>***if answers '1' for at least one above question, proceed with Q 060***				
60	For how long have you been experiencing these symptoms/problems<br><br>ये लक्षण/कठिनाईयाँ आप कब से अनुभव कर रहे हैं	______ **Years**	______ **Months**	
61	Have you ever been treated for these problems/symptoms?<br>क्या कभी आपकी इन समस्याओं/लक्षणों का उपचार हुआ है ?	**Yes (1)**	**No (0)**	If yes, Skip to 63<br><br>यदि हाँ तो 63 पर जाएं
62	Reasons for not seeking treatment:<br>उपचार नहीं करवाने का कारण<br>**(*Multiple responses possible)***	*1=Problem not serious समस्या का गंभीर न होना*<br>*2=Lack of time to visit doctors/clinics due to work काम के कारण चिकित्सक/क्लिनिक के पास जाने के लिए समय का अभाव*<br>*3=Financial difficulties वित्तीय कठिनाईयाँ*<br>*98=Others (specify) अन्य तो स्पष्ट करें*		
63	What is the name of facility/hospital you visited?<br>उस सुविधा/अस्पताल का नाम जिसका लाभ आपने उठाया ?			

64	Ask 'type of provider/facility' for last episode for which treatment was sought. अंतिम बार जब आप ने उपचार का लाभ लिया, **उस प्रदाता/सुविधा का प्रकार**	*Govt. clinic/dispensary* सरकारी क्लिनीक/डिसपेंसरी *=01*<br>*Govt hospital* सरकारी अस्पताल*=02*<br>*Private physician/clinic* निजी चिकित्सक/क्लिनीक *=03*<br>*Private hospital/nursing home (including NGO/trust hospitals)* निजी अस्पताल/नर्सिंग होम(एन.जी.ओ./ट्रस्ट अस्पतालों से सहित) *=04*<br>*Quacks/informal providers* फर्जी चिकित्सक/असंगठित प्रदाता *=05*<br>*Medicine shops/pharmacy/old prescriptions* दवाखाना/फार्मेसी/पूराने नुस्खे *=06*<br>*Hakim/vaids* हकीम/वैद्य *=07*<br>*Other* (specify) अन्य (स्पष्ट करें) =98		
65	Reasons for seeking treatment from the provider/facility mentioned<br>प्रदाता से उपचार/लिखित सुविधा प्राप्त करने का कारण<br>**(*Multiple responses possible)***	Nearest health facility/convenient (including convenient operational hours) निकटतम स्वास्थ्य सुविधा/सूविधाजनक (संचालन के सुविधाजनक घंटे) =01<br>Low cost/financial reasons कम लागत/वित्त =02<br>Usual choice for treatment/past experience of effective treatment उपचार का नियमित विकल्प/अतीत के प्रभावी उपचार का अनुभव =03<br>neighbours/friends advised पड़ोसियों/मित्रों का सलाह =04<br>Others (specify) अन्य(स्पष्ट करें) =98<br>Not sure/DK/CS =99		
66	Where you required to be hospitalized during **last 12 months** for treatment of repiratory ailments/problems you have mentioned?<br>जिन गंभीर बीमारीयों/समस्याओं का आपने उल्लेख किया हैं, उपचार के लिए विगत **12 महीनों में** आपको किसी अस्पताल में भरती होना पड़ा ?	**Yes**<br>**(1)**	**No**<br>**(0)**	**If NO**<br>**Skip to Next Sub-Section**
67	If yes, Name of the hospital यदि हाँ, तो अस्पताल का नाम			
68	If yes, Type of the facility (If not sure leave blank-fill by community interaction)<br>यदि हाँ, तो सुविधा का प्रकार (यदि आश्वस्त नही तो रिक्त छोड़ दें – समुदाय से बातचीत कर भरें)	Govt. clinic/dispensary<br>सरकारी क्लिनीक/डिसपेंसरी =01<br>Govt hospital<br>सरकारी अस्पताल=02<br>Private physician/clinic<br>निजी चिकित्सक/क्लिनीक =03<br>Private hospital/nursing home (including NGO/trust hospitals)<br>निजी अस्पताल/नर्सिंग होम(एन.जी.ओ./ट्रस्ट अस्पतालों से सहित) =04<br>Quacks/informal providers<br>फर्जी चिकित्सक/असंगठित प्रदाता =05<br>Medicine shops/pharmacy/old prescriptions<br>दवाखाना/फार्मेसी/पूराने नुस्खे =06<br>Hakim/vaids हकीम/वैद्य =07<br>Other (specify) अन्य (स्पष्ट करें) =98		
69	Are you currently consuming any medicines/under any treatment for the reported problems/symptoms (If prescription/medical records available, check & write names & medicine providers in a space provided.<br>क्या आप समस्याओं /लक्षणों के निदान के लिए उपचार के तहत इन दिनों दवाईओं का सेवन कर रहे हैं (यदि नुस्खे की प्रति/मेडिकल दस्तावेज उपलब्ध हैं, जाँच कर दिए गए स्थान में नाम/प्रदाता का नाम लिख लें)	**Yes**<br>**(1)**	**No (0)**	Medicine Name<br>1.<br>2.

During last 12 months how much did you spent on the treatment of the reported problems/alignments
(For Respiratory Ailments/Problems)
अब मै आपसे आपके **श्वास संबंधी रोग/समस्या** के उपचार पर हुए खर्च से संबंधित कुछ सूचनाएं संकलित करना चाहूँगा|

Please provide amount spent for<br>कृपया खर्च की गए राशि के बारे में बताएं<br>**(For Respiratory Ailments/Problems)**<br>**श्वास संबंधी रोग/समस्या के लिए**<br>**Col (1)**		Total cost<br>कुल लागत<br>**Col (2)**	Amount paid out-of pocket (own income/savings)<br>दी गई राशि (अपनी आय/बचत)<br>**Col (3)**	Amount from other sources<br>अन्य स्रोतों से राशि<br>**Col (4)**	Source<br>स्रोत<br>**Col (5)**	Amount provided under RSBY scheme<br>आर.एस.बी.वाई. योजना के तहत दी गई राशि<br>**Col (6)**
70	Doctor's fee चिकित्सक का शुल्क					
71	Diagnostic tests निदान के लिए जाँच					
72	Medicine/injections दवाई/सूईयाँ					
73	Bed charges बेड शूल्क					
74	Surgery charges शल्य चिकित्सा शुल्क					
75	Any special diet/other food<br>कोई विशेष आहार/अन्य भोजन					
76	Follow-up costs आगे की जाँच की लागत					
77	Transportation (including ambulance) परिवहन (एम्बुलेंश सहित)					
78	Any other indirect costs (wage loss of attendants etc.)<br>कोई और अप्रत्यक्ष खर्च (सेवक को पारिश्रमिक का नुकसान आदि)					
79	**Total expenses** कुल खर्च					

Instructions: निर्देश
For 3: Ask respondent whether entire amount was met with self-income/salary/from other household member. If so, copy the total costs from Col.2 to Col. 3 and skip col.4 & col.5 उत्तरदाता से पूछें कि क्या पूरी राशि खुद की आय/वेतन/घर के अन्य सदस्य से पूरी की गई। यदि हाँ, कॉलम 4 एवं कॉलम 5 को छोड़ कॉलम 2 से कॉलम 3 में लिखित कुल खर्चे को नकल कर लें।
For 4: Entre the amount that was with supported from other sources: अन्य स्रोतों से प्राप्त कुल राशि को प्रविष्ट कर लें :
If Partial support=include amount यदि आंशिक मदद=राशि शामिल करें
If total (entire amount supported by external sources)=copy Col. 2. यदि योग (वाह्य स्रोत से प्राप्त कुल राशि)=कालम 2 की नकल कर लें
For 5: Other/External sources: अन्य/बाह्य स्रोत :
1=Borrow from friend/neighbor/relatives; मित्रों/पड़ोसियों/रिश्तेदारों से कर्ज
2=Loans from office; दफतर से उधार
3=Paid by the employee कर्मचारी द्वारा भुगतान
4=Loan (with interest) from money lender साहूकार से लिया गया सूद के साथ उधार
98=Others (specific) अन्य (स्पष्ट करें)

80	How were the expenses met? (*Multiple responses possible*)<br><br>खर्चे कैसे पूरे किये गए ? (अनेक उत्तर संभव हैं)<br><br>*Note: These questions are to be asked if the respondent is unable to give break-up of the expenses incurred (and its financing sources)* नोट : ये प्रश्न तब पूछे जाने है *यदि उत्तरदाता खर्च (उसके वित्तीय स्रोत)टुकड़ों में नहीं बता पता है*	*Self savings* खुद की बचत *=1*<br>*Employer paid* नियोक्ता द्वारा देय *=2*<br>*Friends/family* मित्रों / रिश्तेदारों *=3*<br>*Health ins-RSBY* स्वास्थ्य में आर.एस.बी.वाई. *=4*<br>*Health insurance* स्वास्थ्य बीमा *(others* अन्य*)=5*<br>*Borrowings from money lenders with interest* साहूकारों से सूद सहित लिया गया कर्ज *=6*<br>*Selling of assets* संपत्ति बेचकर *=7*<br>*Others* (specify) अन्य *=98*<br>*DK/CS=99*	
81	Was it of any difficulty for your family/yourself to meet the costs associated with your (child's) treatment, and spending the amount you mentioned?<br><br>क्या आपके / आपके परिवार के लिए आपके बच्चे के उपचार पर खर्च, जिसके बारे में आपने उल्लेख किया, करने में किसी तरह की कठिनाई हुई ?	*1=Extremely difficult* अत्याधिक कठिनाई<br>*2=Somewhat difficult* कुछ हद तक कठिनाई<br>*3=Not at all* बिलकुल नहीं	
82	In the last 12 months, how many days you did not go to work/was confined to bed due to these reported problems/difficulties?<br><br>इन सूचित समस्याओं / कठिनाईयों के कारण, विगत **12 महीनों** में आप कितने दिनों तक काम पर नहीं जा सके / बिस्तर पर पड़े रहे ?		
83	Do you work in a dusty environment? (e.g. construction sites, roadside vending, rickshaw-pulling, porters)<br><br>क्या आप धूल भरे परिवेश मे काम करते हैं (जैसे भवन निर्माण स्थल, रोड के किनारे दुकान, रिक्शा खींचना, कुली का काम)	**Yes (1)**	**No (0)**

D. Short-term Morbidities (अल्पकालिक अस्वस्थता) [ONLY FOR RESPONDENTS/ NO PROXY RESPONSE]

(Exclude any disease covered in 'A' & 'B' Sub-Section, and any hospitalization)

(किसी भी कारण से अस्पताल में भर्ती होने एवं भाग A और B में शामिल रोगों को छोड़ दें)

84	Were you/child sick ever, during the last 4 weeks/1month<br>क्या आप या आपका बच्चा पिछले 4 हफते/1 महीने में कभी बीमार हुए/हुआ था ?	**Yes (1)**	**No (0)**	**If NO Skip to Next Sub-Section**
85	Line number of ailing child/self [ADD ADDITIONAL SHEET IN CASE OF RESPONDENT AND CHILD BOTH SUFFER FROM SHORT-TERM MORBIDITIES]<br>बीमार बच्चे/स्वंय की लाइन संख्या			

86	Type of illness<br>बीमारी के प्रकार<br>**[Put ✓ Mark]**<br>***(Multiple responses possible)***	1	fever; बुखार
		2	Cough & cold (with/without fever) सर्दी एवं खाँसी (बुखार के साथ/बिना बुखार के)
		3	Respiratory infections श्वास संबंधी संक्रमण
		4	Lose motion/diarrhoea/dysentery vomiting<br>पतला दस्त/अतिसार/पेचिश/उल्टी
		5	Eye infection आँख का संक्रमण
		6	Chicken pox चेचक
		7	Problems in teeth दाँत की समस्या
		8	Ear infection कान का संक्रमण
		9	Gastric disorders/stomach ache गैस की समस्या/पेट दर्द
		10	Body ache/joint pains शरीर का दर्द/जोड़ों का दर्द
		11	Skin disease/rashes चर्म रोग/दाने निकलना
		98	Others(Specify)_______________________________

87	Duration of illness (in days)<br>बीमारी की अवधि(दिनों में)	
88	No. of days loss in work/school<br>कार्य/स्कूल दिवस का नुकसान (दिनों की संख्या)	
89	Was the illness treated? Yes (1) No (0)<br>क्या बीमारी का उपचार किया गया था ?	
90	What is the name of facility you visited?<br>आपने जिस सुविधा का लाभ लिया उसका नाम क्या है ?	

91	Ask 'type of provider/facility' for last episode for which treatment was sought?<br>आखिरी बार जब आपको यह तकलीफ हुई/बीमार हुए तब आप किसको दिखाने के लिए कहाँ गए थे ?	*Govt. clinic/dispensary* सरकारी क्लिनीक/डिसपेंसरी *=01*
		Govt hospital सरकारी अस्पताल*=02*
		Private physician/clinic निजी चिकित्सक/क्लिनीक *=03*
		Private hospital/nursing home (including NGO/trust hospitals) निजी अस्पताल/नर्सिंग होम(एन.जी.ओ./ट्रस्ट अस्पतालों से सहित) *=04*
		Quacks/informal providers फर्जी चिकित्सक/असंगठित प्रदाता *=05*
		Medicine shops/pharmacy/old prescriptions दवाखाना/फार्मेसी/पूराने नुस्खे *=06*
		Hakim/vaids हकीम/वैद्य *=07*
		Other (specify) अन्य (स्पष्ट करें) =98_______________________________________

92	Reasons for the seeking treatment from the provider mentioned in **Q 91**<br>प्रबंधक से उपचार करवाने का कारण<br>(जिसका उल्लेख **प्रश्न 91** है)<br>**(*Multiple responses possible)***<br>**[Put ✓ Mark]**		*Nearest health facility/convenient (including convenient operational hours)* निकटतम स्वास्थ्य सुविधा/सूविधाजनक (संचालन के सुविधाजनक घंटे) *=01*
			Low cost/financial reasons कम लागत/वित्त *=02*
			Usual choice for treatment/past experience of effective treatment उपचार का नियमित विकल्प/अतीत के प्रभावी उपचार का अनुभव *=03*
			neighbours/friends advised पड़ोसियों/मित्रों का सलाह *=04*
			taken by police/other persons/ambulance पुलिस/अन्य व्यक्तियों/एमबुलेंस द्वारा*=05*
			Others (specify)*= 98*
			Not sure/DK/CS)=99

93	Are you/child **still** sick/suffering from the disease/problem you have mentioned<br>क्या आप/आपका बच्चा **आज भी** उस रोग/समस्या, जिसका उल्लेख आपने किया है, से पीड़ित है ?	**Yes -1**	**No-0**
94	Was any other provider consulted for the treatment?<br>क्या उपचार के लिए किसी अन्य प्रदाता का भी परामर्श लिया गया था ? *(2nd Recourse)*	**Yes -1**	**No -0**

95	If yes, type of provider<br>यदि हाँ, तो प्रदाता का प्रकार<br>**(*Multiple responses possible)***<br>**[Put ✓ Mark]**		(Govt. dispensary/clinic सरकारी डिसपेंसरी/क्लीनिक =1
			Govt. hospital OPD सरकारी अस्पताल का ओ.पी.डी. =2
			Private physicians' clinics निजी चिकित्सकों का क्लीनिक =3
			Pvt. Nursing homes निजी नर्सिंग होम =4
			Pharmacy (OTC) फार्मेसी ओ.टी.सी. =5
			Quacks/informal providers फर्जी/असंगठित प्रदाता =6
			Traditional practitioners (hakims/vaids) पारंपरिक प्रदाता जैसे हकीम/वैद्य =7
			Others (specify) अन्य(स्पष्ट करें) =98

What was the total amount spent for the treatment and related expenses for the disease(s) mentioned (**Short-term Morbidities** अल्पकालिक अस्वस्थता), during the **past 1 Months (1 महीने में)**

	Please provide amount spent for<br>कृपया खर्च की गए राशि के बारे में बताएं<br>**Col (1)**<br>**(For Short-term Morbidities)**<br>**अल्पकालिक अस्वस्थता के लिए**	Total cost<br>कुल लागत<br>**Col (2)**	Amount paid out-of pocket (own income/savings)<br>दी गई राशि (अपनी आय/बचत)<br>**Col (3)**	Amount from other sources<br>अन्य स्रोतों से राशि<br>**Col (4)**	Source<br>स्रोत<br>**Col (5)**	Amount provided under RSBY scheme आर. एस.बी.वाई.योजना के तहत दी गई राशि<br>**Col (6)**
96	Doctor's fee<br>चिकित्सक का शुल्क					
97	Diagnostic tests<br>निदान के लिए जाँच					
98	Medicine/injections<br>दवाई/सूईयाँ					
99	Any special diet/other food<br>कोई विशेष आहार/अन्य भोजन					
100	Follow-up costs<br>आगे की जाँच की लागत					
101	Transportation (including ambulance)<br>परिवहन (एम्बुलेंश सहित)					
102	Any other indirect costs (wage loss of attendants etc.) कोई और अप्रत्यक्ष खर्च (सेवक को पारिश्रमिक का नुकसान आदि)					
103	**Total expenses**<br>**कुल खर्च**					

Instructions: निर्देश
For 3: Ask respondent whether entire amount was met with self-income/salary/from other household member. If so, copy the total costs from Col.2 to Col. 3 and skip col.4 & col.5
उत्तरदाता से पूछें कि क्या पूरी राशि खुद की आय/वेतन/घर के अन्य सदस्य से पूरी की गई। यदि हाँ, कॉलम 4 एवं कॉलम 5 को छोड़ कॉलम 2 से कॉलम 3 में लिखित कुल खर्चे को नकल कर लें।
For 4: Entre the amount that was with supported from other sources:
अन्य स्रोतों से प्राप्त कुल राशि को प्रविष्ट कर लें :
If Partial support=include amount यदि आंशिक मदद=राशि शामिल करें
If total (entire amount supported by external sources)=copy Col. 2.
यदि योग (वाह्य स्रोत से प्राप्त कुल राशि)=कालम 2 की नकल कर लें
For 5: Other/External sources: अन्य/बाह्य स्रोत :
1=Borrow from friend/neighbor/relatives; मित्रों/पड़ोसियों/रिश्तेदारों से कर्ज
2=Loans from office; दफतर से उधार
3=Paid by the employee कर्मचारी द्वारा भुगतान
4=Loan (with interest) from money lender साहूकार से लिया गया सूद के साथ उधार
98=Others (specific) अन्य (स्पष्ट करें)

104	How were the expenses met?<br>*Note: This questions are to be asked if the respondent is unable to give break-up of the expenses incurred (and its financing sources)*<br>नोट : ये प्रश्न तब पूछे जाने है *यदि उत्तरदाता खर्च (उसके वित्तीय स्रोत)टुकड़ों में नहीं बता पता है*<br>खर्चे कैसे पूरे किये गए ?<br>**(*Multiple responses possible)***<br>**(अनेक उत्तर संभव हैं)**	*1*	*Self savings* खुद की बचत
		2	*Employer paid* नियोक्ता द्वारा देय
		3	*Friends/family* मित्रों/रिश्तेदारों
		4	*Health ins-RSBY* स्वास्थ्य में आर.एस.बी.वाई.
		5	*Health insurance* स्वास्थ्य बीमा *(others* अन्य*)*
		6	*Borrowings from money lenders with interest* साहूकारों से सूद सहित लिया गया कर्ज
		7	*Selling of assets* संपत्ति बेचकर
		98	*Others* (specify) अन्य_______________
		99	*DK/CS=99*

105	Was it of any difficulty for your family/yourself to meet the costs associated with your (child's) treatment, and spending the amount you mentioned?<br><br>क्या आपके/आपके परिवार के लिए आपके बच्चे के उपचार पर खर्च, जिसके बारे में आपने उल्लेख किया, करने में किसी तरह की कठिनाई हुई ?	Extremely difficult अत्याधिक कठिनाई	1
		Somewhat difficult कुछ हद तक कठिनाई	2
		Not at all बिलकुल नहीं	3
106	In the last 12 months, how many days you did not go to work/was confined to bed due to these reported problems/difficulties?<br><br>इन सूचित समस्याओं/कठिनाईयों के कारण, विगत **12 महीनों** में आप कितने दिनों तक काम पर नहीं जा सके/बिस्तर पर पड़े रहे ?		

E. Chronic Morbidities (Only for ADULTS above 16 years) दीर्घकालिक अस्वस्थता (केवल 16 वर्ष से अधिक उम्र के वयस्कों के लिए)

[ONLY FOR RESPONDENTS/ NO PROXY RESPONSE]

107	Were you ever diagnosed with/did any doctor ever tell you that you have any of these diseases<br><br>क्या आपको कभी निम्नांकित रोगों से पीड़ित पाया गया/क्या क्भी किसी चिकित्सक ने आपको निम्नलिखित रोगों से पीड़ित बताया ?	**Yes (1)**	**No (0)**	**If No Skip to NEXT Sub-Section**
108	**Disease Index:** सूचकांक [ADD ADDITIONAL SHEET IN CASE OF MULTIPLE MORBIDITIES]<br><br>D1 – Diabetes; मधुमेह<br>D2 – Heart diseases (including stroke, CHD, angina); दिल की बीमारियाँ (हृदयाघात, सी.एच.डी., एनजाइना)<br>D3 – Hypertension; रक्तचाप<br>D4 – Chronic respiratory ailments (COPD, CB, Asthma); दीर्घकालिक श्वासप्रणाली का रोग<br>D5 – Cancers; कैंसर<br>Other-Other Diseases			
109	How did you come to know about the disease?<br>आपको रोग के बारे में कैसे पता लगा ? *(Medical diagnosis मीडिया से=1; Self स्वयं=2; Relatives रिश्तेदारों से =3; Friends मित्रों से=4; Others (Specify) अन्य से – स्पष्ट करें=98)*			
110	When was this disease first diagnosed रोग की पहचान पहली बार की कब हुई ?<br>(Put months and years as: january, 2012=0112)			
111	Is any medical treatment taken for the ailment<br>क्या रोग का कोई चिकित्सकीय उपचार किया गया ? *(Yes -1, No -0 DK/CS=99)*			
112	If Q 111=0: Reasons for not seeking treatment उपचार नहीं करवाने का कारण			
113	What is the name of facility you visited?<br>आपने जिस सुविधा का लाभ लिया उसका नाम क्या है ?			

114	Ask ‘type of provider/facility’ for last episode for which treatment was sought?<br>आखिरी बार जब आपको यह तकलीफ हुई/बीमार हुए तब आप किसको दिखाने के लिए कहाँ गए थे ?	1	*Govt. clinic/dispensary* सरकारी क्लिनीक/डिसपेंसरी
		2	*Govt hospital* सरकारी अस्पताल
		3	*Private physician/clinic* निजी चिकित्सक/क्लिनीक
		4	*Private hospital/nursing home (including NGO/trust hospitals)* निजी अस्पताल/नर्सिंग होम(एन.जी.ओ./ट्रस्ट अस्पतालों से सहित)
		5	*Quacks/informal providers* फर्जी चिकित्सक/असंगठित प्रदाता
		6	*Medicine shops/pharmacy/old prescriptions* दवाखाना/फार्मेसी/पूराने नुस्खे
		7	*Hakim/vaids* हकीम/वैद्य
		98	*Other* (specify) अन्य (स्पष्ट करें)
115	Reasons for the seeking treatment from the provider mentioned in **Q 114**<br><br>प्रबंधक से उपचार करवाने का कारण (जिसका उल्लेख **प्रश्न 114** में है)<br><br>**(*Multiple responses possible)***	1	(*Nearest health facility/convenient (including convenient operational hours)* निकटतम स्वास्थ्य सुविधा/सूविधाजनक (संचालन के सुविधाजनक घंटे)
		2	*Low cost/financial reasons* कम लागत/वित्त
		3	*Usual choice for treatment/past experience of effective treatment* उपचार का नियमित विकल्प/अतीत के प्रभावी उपचार का अनुभव
		4	*neighbours/friends advised* पड़ोसियों/मित्रों का सलाह
		5	*taken by police/other persons/ambulance* पुलिस/अन्य व्यक्तियों/एमबुलेंस द्वारा
		98	*98=Others* (specify)________________
		99	*99=Not sure/DK/CS*

116	Current status of treatment उपचार की ताजा स्थिति<br><br>(Treatment ongoing इलाज चल रहा है=1; Treatment discontinued इलाज रोक दिया गया =2; No treatment, but medicines taken SOS कोई ईलाज नहीं, लेकिन एस.ओ.एस. के रूप में दवाईयाँ ली गईं =3; Others (specify) अन्य (स्पष्ट करें) =98)		
117	Source of medicines<br>दवाईयों का स्रोत	*Govt. clinic/dispensary* सरकारी क्लिनीक / डिसपेंसरी *=01*<br>*Govt hospital* सरकारी अस्पताल*=02*<br>*Private physician/clinic* निजी चिकित्सक / क्लिनीक *=03*<br>*Private hospital/nursing home (including NGO/trust hospitals)* निजी अस्पताल / नर्सिंग होम(एन.जी.ओ. / ट्रस्ट अस्पतालों से सहित) *=04*<br>*Quacks/informal providers* फर्जी चिकित्सक / असंगठित प्रदाता *=05*<br>*Medicine shops/pharmacy/old prescriptions* दवाखाना / फार्मेसी / पूराने नुस्खे *=06*<br>*Hakim/vaids* हकीम / वैद्य *=07*<br>*Other* (specify) अन्य (स्पष्ट करें) =98	
118	Source for diagnostic tests/check-ups<br><br>निदान के लिए जाँच / मेडिकल जाँच का स्रोत	*Govt. clinic/dispensary* सरकारी क्लिनीक / डिसपेंसरी *=01*<br>*Govt hospital* सरकारी अस्पताल*=02*<br>*Private physician/clinic* निजी चिकित्सक / क्लिनीक *=03*<br>*Private hospital/nursing home (including NGO/trust hospitals)* निजी अस्पताल / नर्सिंग होम(एन.जी.ओ. / ट्रस्ट अस्पतालों से सहित) *=04*<br>*Quacks/informal providers* फर्जी चिकित्सक / असंगठित प्रदाता *=05*<br>*Medicine shops/pharmacy/old prescriptions* दवाखाना / फार्मेसी / पूराने नुस्खे *=06*<br>*Hakim/vaids* हकीम / वैद्य *=07*<br>*Other* (specify) अन्य (स्पष्ट करें) =98	
119	Where you required to be hospitalized during **last 12 months** for treatment of any of the chronic illness/problems you have mentioned?<br>आपको किस अस्पताल में, जिन गंभीर बीमारीयों / समस्याओं का आपने उल्लेख किया हैं, उपचार के लिए विगत **12 महीनों** में में भरती होना पड़ा ?	**Yes (1)**	**No (0)**
120	If yes, Name of the hospital यदि हाँ, तो अस्पताल का नाम		
121	If yes, Type of the facility (If not sure leave blank-fill by community interaction) यदि हाँ, तो सुविधा का प्रकार (यदि आश्वस्त नहीं है तो खाली छोड़ दें – समुदाय से बातचीत करें भरें)	*Govt. clinic/dispensary* सरकारी क्लिनीक / डिसपेंसरी *=01*<br>*Govt hospital* सरकारी अस्पताल*=02*<br>*Private physician/clinic* निजी चिकित्सक / क्लिनीक *=03*<br>*Private hospital/nursing home (including NGO/trust hospitals)* निजी अस्पताल / नर्सिंग होम(एन.जी.ओ. / ट्रस्ट अस्पतालों से सहित) *=04*<br>*Quacks/informal providers* फर्जी चिकित्सक / असंगठित प्रदाता *=05*<br>*Medicine shops/pharmacy/old prescriptions* दवाखाना / फार्मेसी / पूराने नुस्खे *=06*<br>*Hakim/vaids* हकीम / वैद्य *=07*<br>*Other* (specify) अन्य (स्पष्ट करें) =98	

During **last 12 months** how much did you spent on the treatment of the reported problems/alignments
(For Chronic Morbidities दीर्घकालिक अस्वस्थता **के लिए)**

122	Please provide amount spent for कृपया खर्च की गए राशि के बारे में बताएं **(For Chronic Morbidities)** दीर्घकालिक अस्वस्थता **के लिए** **Col (1)**	Total cost कुल लागत **Col (2)**	Amount paid out-of pocket (own income/savings) दी गई राशि (अपनी आय / बचत) **Col (3)**	Amount from other sources अन्य स्रोतों से राशि **Col (4)**	Source स्रोत **Col (5)**	Amount provided under RSBY scheme आर.एस.बी.वाई.योजना के तहत दी गई राशि **Col (6)**
123	Doctor's fee चिकित्सक का शुल्क					
124	Diagnostic tests निदान के लिए जाँच					
125	Medicine/injections दवाई / सूईयाँ					
126	Bed charges बेड शूल्क					
127	Surgery charges शल्य चिकित्सा शुल्क					
128	Any special diet/other food कोई विशेष आहार / अन्य भोजन					
129	Follow-up costs आगे की जाँच की लागत					
130	Transportation (including ambulance) परिवहन (एम्बुलेंश सहित)					
131	Any other indirect costs (wage loss of attendants etc.) कोई और अप्रत्यक्ष खर्च (सेवक को पारिश्रमिक का नुकसान आदि)					
132	**Total expenses** कुल खर्च					

Instructions: निर्देश
***For 3:** Ask respondent whether entire amount was met with self-income/salary/from other household member. If so, copy the total costs from Col.2 to Col. 3 and skip col.4 & col.5 उत्तरदाता से पूछें कि क्या पूरी राशि खुद की आय / वेतन / घर के अन्य सदस्य से पूरी की गई। यदि हाँ, कॉलम 4 एवं कॉलम 5 को छोड़ कॉलम 2 से कॉलम 3 में लिखित कुल खर्चे को नकल कर लें।*
***For 4:** Entre the amount that was with supported from other sources: अन्य स्रोतों से प्राप्त कुल राशि को प्रविष्ट कर लें :If Partial support=include amount यदि आंशिक मदद=राशि शामिल करें If total (entire amount supported by external sources)=copy Col. 2. यदि योग (वाह्य स्रोत से प्राप्त कुल राशि)=कालम 2 की नकल कर लें*
***For 5:** Other/External sources: अन्य / बाह्य स्रोत :1=Borrow from friend/neighbor/relatives; मित्रों / पड़ोसियों / रिश्तेदारों से कर्ज2=Loans from office; दफतर से उधार 3=Paid by the employee कर्मचारी द्वारा भुगतान4=Loan (with interest) from money lender साहूकार से लिया गया सूद के साथ उधार9=Others (specific)अन्य (स्पष्ट करें)*

133	How were the expenses met? **(Multiple responses possible)** खर्चे कैसे पूरे किये गए ? **(अनेक उत्तर संभव हैं)** *Note: These questions are to be asked if the respondent is unable to give break-up of the expenses incurred (and its financing sources)* नोट : ये प्रश्न तब पूछे जाने है *यदि उत्तरदाता खर्च (उसके वित्तीय स्रोत)टुकड़ों में नहीं बता पता है*	*Self savings* खुद की बचत *=1*<br>*Employer paid* नियोक्ता द्वारा देय *=2*<br>*Friends/family* मित्रों / रिश्तेदारों *=3*<br>*Health ins-RSBY* स्वास्थ्य में आर.एस.बी.वाई. *=4*<br>*Health insurance* स्वास्थ्य बीमा *(others* अन्य*)=5*<br>*Borrowings from money lenders with interest* साहूकारों से सूद सहित लिया गया कर्ज *=6*<br>*Selling of assets* संपत्ति बेचकर *=7*<br>*Others* (specify) अन्य *=98*<br>*DK/CS=99*
134	Was it of any difficulty for your family/yourself to meet the costs associated with your (child's) treatment, and spending the amount you mentioned? क्या आपके / आपके परिवार के लिए आपके बच्चे के उपचार पर खर्च, जिसके बारे में आपने उल्लेख किया, करने में किसी तरह की कठिनाई हुई ?	*1=Extremely difficult* अत्याधिक कठिनाई<br>*2=Somewhat difficult* कुछ हद तक कठिनाई<br>*3=Not at all* बिलकुल नहीं

F. Accidental Injury, Treatment and Financing [INCLUDE ANY HOUSEHOLD MEMBER/PROXY ALLOWED]

**F1**	Did you/any household member suffer from any accidents or an injury in the past 12 months?<br>(Ignore minor cuts/bruises; include those that required medical treatment)<br>क्या आप/आपके परिवार का कोई सदस्य विगत 12 महीनों में किसी दुर्घटना या या चोट का शिकार हुआा ? (छोटे आकार के कटने/छिलने को नजर अंदाज कर दें ; उनको शामिल करे जिनके लिए चिकित्सकीय उपचार वांछित है)	**Yes (1)**	**No (0)**	If no, Skip to next Sub-Section<br>यदि नहीं,तो अगले भाग में जाएं
135	Indicate which members suffered such injuries<br>(Only include currently surviving members; for dead members record in Block___): [ADD ADDITIONAL SHEET IN CASE OF MORE THAN TWO] (Instruction – copy Member Line Number from Block-___)<br>उन सदस्यों को इंगित करें जिन्हें चोटे आईं<br>(**केवल जीवित सदस्यों को ही शामिल करे** ; मृत सदस्यों को ब्लॉक में अंकित करें ——) (निर्देश – सदस्य का लाइन नंबर ब्लॉक से नकल करें)	**ID-1**		**ID-2**
136	Type of accident/Mechanism of Injury<br>दुर्घटना का प्रकार/चोट लगने का तरीका<br>(*1=motor vehicle* मोटर गाड़ी, *2=pedestrian-vehicle crash* पैदल चलने वालों और वाहनों के बीच टक्कर, *3=motorcycle* मोटर साईकल, *4=bicycle* साईकल, *5=fall* गिरना, *6=gunshot/firearms* बंदूक की गोली/आग्नेयास्त्र, *7=stab/cuts* घोपने/कटने, *8=fire/burn s* आग लगने/जलने, *9=poisoning* जहर देने, *10=drowning* डूबने, *11=others*, *98=Others* (specify) अन्य (स्पष्ट करें); *99=DK/CS*)			
137	Where did the injury occur?<br>चोट किस स्थान पर लगी ?<br><br>(*1=home* घर पर, *2=school* स्कूल में, *3=streets/highways/railways* गलियों में/ उच्चमार्ब पर/रेलस्टेशन, *4=trade & service areas (shops, offices)* व्यापार एवं सेवा क्षेत्र (दुकान, दफतर), *5=water bodies* जलस्थलों, *6=industrial/construction areas* ओद्योगिक/निर्माण क्षेत्रों में, *98=others (specify)* अन्य (स्पष्ट करें); ; *99=DK/CS*)			
138	When (in the last 12 months) did the accident happen?<br>पिछले 12 महीनों में दुर्घटना कब घटी ?<br>(*1=within last 30 days* पिछले 30 दिनों के अंदर , *2=last 3 months* पिछले 3 मिहीनों के अंदर, *3=3-6 months* पिछले 3 से 6 महीनों के अंदर, *4=6-12 months* पिछले 4 से 6 महीनों के अंदर, *98=Others* (specify) अन्य (स्पष्ट करें) *; 99=DK/CS*)			
139	Which body parts/organs were injured/affected by the accident?<br>छुर्घटना/चोट से शरीर का कौन सा भाग/अंग प्रभावित हुआ ?<br>(Head सिर =1; Limb हाथ–पाँव =2; Chest छाती =3; Shoulder/neck/back कंधा/गर्दन/पीठ =4; Internal organ अंदरूनी अंग =5)			
140	Did any other family members/friends accompany you/member at the time of the accident/injury?<br>दुर्घटना/चोट के समय क्या आपके परिवार के सदस्य/मित्र आपके/आपके परिवार के सदस्य के साथ थे ? (*0=No, 1=Yes, 99=DK/CS*)			
141	How you /your family did come to know about the accident/injury of the member?<br>आप /आपके परिवार को सदस्य के दुर्घटना की जानकारी कैसे मिली ?<br>(*1=police* पुलिस, *2=hospital authorities* अस्पताल के अधिकारियों, *3=friends/neighbors* मित्रों/पड़ोसियों, *4=unknown strangers* अनजान लोगों से *; 98=Others* (specify) अन्य (स्पष्ट करें)			
142	Did you/member receive any medical attention/treatment at the accident/injury site? क्या आपको/आपके परिवार के सदस्य को दुर्घटना/चोट लगने की जगह पर कोई चिकित्सकीय घ्यान/उपचार मिला ?<br>(*0=No, 1=Yes, 99=DK/CS*)			

		ID-1	ID-2
143	Were you/member taken/went to any hospital/clinic to receive treatment after the injury/accident?<br>दुर्घटना/चोट के बाद क्या आप/आपके सदस्य को उपचार के लिए किसी अस्पताल/क्लिनिक ले जाया गया/आप उपचार के लिए गए ? (*0=No, 1=Yes, 99=DK/CS*)		
144	Name of the hospital<br><br>अस्पताल का नाम		
145	Type of the facility<br>सुविधा का प्रकार<br>*Govt. clinic/dispensary* सरकारी क्लिनीक/डिसपेंसरी *=01;*<br>*Govt hospital* सरकारी अस्पताल*=02;*<br>*Private physician/clinic* निजी चिकित्सक/क्लिनीक *=03;*<br>*Private hospital/nursing home (including NGO/trust hospitals)* निजी अस्पताल/नर्सिंग होम(एन.जी.ओ./ट्रस्ट अस्पतालों से सहित) *=04;*<br>*Quacks/informal providers* फर्जी चिकित्सक/असंगठित प्रदाता *=05;*<br>*Medicine shops/pharmacy/old prescriptions* दवाखाना/फार्मेसी/पूराने नुस्खे *=06;*<br>*Hakim/vaids* हकीम/वैद्य *=07;*<br>*Other* (specify) अन्य (स्पष्ट करें) =98		
146	How were you/member taken/went to the hospital/clinic/physician mentioned in Q 198? आप /आपके सदस्य को उस अस्पताल/क्लीनिक/चिकित्सक जिसका उल्लेख प्रश्न 198 में किया गया है कैसे ले जाया गया/गये ?<br>(*1=Govt ambulance* सरकारी एम्बुलेंस*, 2=pvt. Ambulance* निजी एम्बुलेंस*, 3=pvt vehicles* निजी गाड़ी *4=police vehicles* पुलिस गाड़ी *5=rickshaw/vans* रिक्शा/वैन*, 98= Others* (specify) अन्य (स्पष्ट करें) ; *99=DK/CS*)		
147	Are you aware of the CATS ambulance service OR the helpline number <u>102/1099</u> for emergency medical transportation service of the Delhi Government? क्या आप दिल्ली सरकार की कैट्स एम्बुलेंस सेवा या आपातकालीन चिकित्सा परिवहन सेवा के लिए जारी हेल्पलाइन नंबर 102/1099 से परिचित हैं ?<br>(*0=No, 1=Yes, 98= Others* (specify); *99=DK/CS*)		
148	Reasons for seeking care/treatment from the particular facility?<br>सुविधा विशेष के तहत देखभाल/उपचार चाहने का कारण<br><br>(*Nearest health facility/convenient (including convenient operational hours)* निकटतम स्वास्थ्य सुविधा/सूविधाजनक (संचालन के सुविधाजनक घंटे) *=01;*<br>*Low cost/financial reasons* कम लागत/वित्त *=02;*<br>*Usual choice for treatment/past experience of effective treatment* उपचार का नियमित विकल्प/अतीत के प्रभावी उपचार का अनुभव *=03;*<br>*neighbours/friends advised* पड़ोसियों/मित्रों का सलाह*=04*<br>*taken by police/other persons/ambulance* पुलिस/अन्य व्यक्तियों/एमबुलेंस द्वारा*=05;*<br>*98=Others* (specify);<br>*99=Not sure/DK/CS*		
149	Did you/member receive immediate treatment (including examination, dressing of the wound, applying bandages/casts) after arriving at the health facility?<br>स्वास्थ्य केन्द्र पर पहुँचने के बाद आप/आपके सदस्य को तत्काल उपचार (जिसमें जाँच, घाव की मरहमपट्टी, बैण्डेज लगाना शामिल है) प्राप्त हुआ ?<br>(*0=No, 1=Yes, 99=DK/CS*)		

		ID-1	ID-2
150	Were any diagnostic tests/scans conducted<br>क्या निदान के लिए कोई जाँच/स्कैन किया गया ? (*0=No, 1=Yes, 99=DK/CS*)		
151	How satisfied were you by the overall quality of treatment/care received while being treated for the accident/injury at the health facility?<br>*स्वास्थ्य केन्द्र पर दुर्घटना/चोट के लिए प्राप्त उपचार की गुणवत्ता से कुल मिलाकर आप कितने संतुष्ट थे ?*<br>*Rate in a scale of 1-5, with 1=Extremely dissatisfied and 5=Very Satisfied*<br>*1 से 5 के स्केल पर रेट करें जिसमें 1=अति संतुष्ट और 5=बहुत संतुष्ट*		
152	Did the accident/injury require any hospitalization/in-patient stay of the member?<br>क्या दुर्घटना/चोट के लिए अस्पताल में भर्ती/रोगी के रूप में सदस्य का रूकना जरूरी था ? (*0=No, 1=Yes, 99=DK/CS*)		
153	Days of stay<br>कितने दिनों तक रूके		
154	Total duration of treatment (include hospitalizations and any follow-up visits) उपचार की कुल अवधि (अस्पताल में भर्ती और बाद के जाँच के लिए अस्पताल जाने को लेकर)		

Total expenditure incurred on account of treatment and related expenses due to the accident/injury of the member in last 12 months? पिछले 12 महीनों में दुर्घटना/चोट के उपचार एवं उससे संबंधित कुल कितना खर्च हुआ ?

	Please provide amount spent for<br>कृपया खर्च की गए राशि के बारे में बताएं<br>**For Accidental Injury**<br>**दुर्घटना/चोट के उपचार के लिए**<br>**Col (1)**	Total cost<br>कुल लागत<br>**Col (2)**	Amount paid out-of pocket (own income/savings)<br>दी गई राशि (अपनी आय/बचत)<br>**Col (3)**	Amount from other sources<br>अन्य स्रोतों से राशि<br>**Col (4)**	Source<br>स्रोत<br>**Col (5)**	Amount provided under RSBY scheme आर. एस.बी.वाई. योजना के तहत दी गई राशि<br>**Col (6)**
155	Doctor's fee<br>चिकित्सक का शुल्क					
156	Diagnostic tests<br>निदान के लिए जाँच					
157	Medicine/injections<br>दवाई/सूईयाँ					
158	Bed charges<br>बेड शूल्क					
159	Surgery charges<br>शल्य चिकित्सा शुल्क					
160	Any special diet/other food<br>कोई विशेष आहार/अन्य भोजन					
161	Follow-up costs<br>आगे की जाँच की लागत					
162	Transportation (including ambulance)<br>परिवहन (एम्बुलेंश सहित)					
163	Any other indirect costs (wage loss of attendants etc.)<br>कोई और अप्रत्यक्ष खर्च (सेवक को पारिश्रमिक का नुकसान आदि)					
164	**Total expenses**<br>कुल खर्च					

Instructions: निर्देश

For 3: Ask respondent whether entire amount was met with self-income/salary/from other household member. If so, copy the total costs from Col.2 to Col. 3 and skip col.4 & col.5

उत्तरदाता से पूछें कि क्या पूरी राशि खुद की आय/वेतन/घर के अन्य सदस्य से पूरी की गई। यदि हाँ, कॉलम 4 एवं कॉलम 5 को छोड़ कॉलम 2 से कॉलम 3 में लिखित कुल खर्चे को नकल कर लें।

For 4: Entre the amount that was with supported from other sources:

अन्य स्रोतों से प्राप्त कुल राशि को प्रविष्ट कर लें :

If Partial support=include amount यदि आंशिक मदद=राशि शामिल करें

If total (entire amount supported by external sources)=copy Col. 2.

यदि योग (वाह्य स्रोत से प्राप्त कुल राशि)=कालम 2 की नकल कर लें

For 5: Other/External sources:

अन्य/बाह्य स्रोत :

1=Borrow from friend/neighbor/relatives; मित्रों/पड़ोसियों/रिश्तेदारों से कर्ज

2=Loans from office; दफतर से उधार

3=Paid by the employee कर्मचारी द्वारा भुगतान

4=Loan (with interest) from money lender साहूकार से लिया गया सूद के साथ उधार

9=Others (specific) अन्य (स्पष्ट करें)

		ID-1	ID-2
165	What has been the major source of finance for the treatment?<br>(*Self savings* खुद की बचत *=1,*<br>*Employer paid* नियोक्ता द्वारा देय *=2,*<br>*Friends/family* मित्रों/रिश्तेदारों *=3,*<br>*Health ins-RSBY* स्वास्थ्य बीमा– आर.एस.बी.वाई. *=4,*<br>*Health insurance* स्वास्थ्य बीमा *(others* अन्य*)=5,*<br>*Borrowings from money lenders with interest* साहूकारों से सूद सहित लिया गया कर्ज *=6,*<br>*Selling of assets* संपत्ति बेचकर *=7,*<br>*Others* अन्य *=8, Others* (specify)*=98;*<br>*DK/CS=99*)		
166	Was it of any difficulty for your family/yourself to meet the costs associated with your (child's) treatment, and spending the amount you mentioned?<br>क्या आपके/आपके परिवार के लिए आपके बच्चे के उपचार पर खर्च, जिसके बारे में आपने उल्लेख किया, करने में किसी तरह की कठिनाई हुई ?<br>*Extremely difficult* अत्याधिक कठिनाई-1<br>*Somewhat difficult* कुछ हद तक कठिनाइ-2<br>*Not at all* बिलकुल नहीं-3		

167	Are you/hh member restricted in any of the following areas as a result of this accident / injury?<br>क्या इस दुर्धटना/चोट के कारण आप/आपके सदस्य को निम्नलिखत क्षेत्रों में से कहीं भी आना जाना बाधित हुआ है ?	For ID-1			For ID-2		
	A. Attending work on a full-time/pre-accident/injury leaves<br>पूरे समय काम पर रहना /दुर्घटना के पूर्व/चोट के कारण अवकाश	**Yes (1)**	**No (2)**	**DK/CS (99)**	**Yes (1)**	**No (2)**	**DK/CS (99)**
	B. Attending school regularly<br>नियमित रूप से स्कूल जाना	**Yes (1)**	**No (2)**	**DK/CS (99)**	**Yes (1)**	**No (2)**	**DK/CS (99)**
	C. Attend daily living activities (bathing/driving/walking/using toilets) दैनिक जीवन की क्रियाओं में भाग लेना (स्नान करना/ड्राइविंग करना/टहलना/शौचालय जाना)	**Yes (1)**	**No (2)**	**DK/CS (99)**	**Yes (1)**	**No (2)**	**DK/CS (99)**
	D. Attend daily household chores (outing/shopping/managing household finance etc.)<br>रोज के घरेलू कार्यों में हिस्सा लेना(जैसे बाहर भ्रमण पर जाना/बाजार जाना/घर के वित्त का प्रबंधन आदि.)	**Yes (1)**	**No (2)**	**DK/CS (99)**	**Yes (1)**	**No (2)**	**DK/CS (99)**

		ID-1	ID-2
168	Have you/hh member missed work due to this accident / injury?<br>क्या आप/आपके घर के सदस्य दुर्घटना/चोट के कारण काम पर नहीं जा सके ?<br>(*0=No, 1=Yes, 99=DK/CS*)		
169	Number of days lost/confined to bed:<br>कुल कितने दिनों तक काम पर नहीं जा सके/बेड तक सीमित रहे		
170	Did the accident/injury cause any permanent/semi-permanent disability?<br>क्या दुर्घटना/चोट के कारण कोई स्थायी/अर्द्ध–स्थायी विकलांगता हुई ?<br>(loss of limb, eyesight, hearing. Cognition etc) (शरीर के अंग, आँखों की रोशनी, श्रवण शक्ति, ज्ञान का खोना) (*0=No, 1=Yes, 98= Others* (specify); *99=DK/CS*)		
171	How difficult you found to adjust with the loss of work / financial hardships, following the accident? दुर्घटना के बाद आपके लिए काम के न रहने से/वित्तीय कठिनाईयों से तालमेल बनाना कितना कठिन हुआ ?<br>*Rate in a scale of 1-3, with 1=Extremely difficult;2=Somewhat difficult; Not difficult at all=3*<br>1 से 3 के स्केल पर रेट करें जिसमें 1=अति कठिन; 2=कुछ हद तक कठिन ; बिलकुल कठिन नहीं=3 है		
172	How would you rate the **current** health status/condition of the member, with respect to the accident/injury<br>*Rate in a scale of 1-5, with Very poor=1; Poor=2; Somewhat OK/moderate=3; Good=4 and 5=Excellent*<br>दुर्घटना/चोट के संदर्भ में आप सदस्य के **मौजूदा स्वास्थ्य** का कैसे मूल्यांकन करेंगे।<br>1 से 5 के स्केल पर मूल्यांकन करें जहाँ बहुत खराब=1 ; खराब=2; कुछ हद तक ठीक/औसत=3; अच्छा=4; बढ़िया=5 है		

G. Other Hospitalizations अन्य अस्पताल में भर्ती (घर के सभी सदस्यों के संबंध में)

[INCLUDE ALL HOUSEHOLD MEMBERS/PROXY ALLOWED]

173	SCREENER CHECK:<br><br>I would want to check again, that apart from the incidents/illnesses/health conditions described by you earlier, were – ANY OTHER HOUSEHOLD MEMBER REQUIRED TO BE HOSPITALIZED FOR ANY DISEASE/ AILMENT, DURING THE LAST OBE YEAR (SINCE LAST HOLI) ailment<br><br>**Interviewer: Please conform that the hospitalization being reported is not due to any accident/injury. If due to 'Accident/Injury' – Go back to Sub-Section E**<br>मैं फिर से जानना चाहूँगा कि आपने जिन घटनाओं/बीमारियों/स्वास्थ्य स्थिति के बारे में पहले बताया है उनको छोड़ क,या परिवार के किसी अन्य सदस्य को पिछले होली के बाद के एक बर्ष के अंदर किसी बीमारी/रोग के लिए अस्पताल में भर्ती करने की जरूरत पड़ी ?<br>**साक्षत्कार कर्त्ता : कृपया ध्यान दें कि जिस कारण से अस्पताल में भर्ती के बारे में बताया जा रहा है, वह दुर्घटना/चोट नहीं है। यदि भर्ती का कारण दुर्घटना/चोट है तो वापस भाग E देखें**	**Yes (1)**	**No (2)**	**If NO Skip to Next Section**
174	Copy the line number for hospitalized member in the next row to the right from Section I भाग 1 के दाहिनी ओर की अगली पंक्ति में जिन सदस्य की भर्ती हुई है उनका लाइन नंबर लिख लें।	MID #1	MID #2	MID #3
175	Line number of hospitalized member अस्पताल में भर्ती सदस्य का लाईन नंबर			
176	Disease code (See code list attached) बीमारी का सांकेतिक नंबर (संलग्न सांकेतिक सूची देखें)			
177	Type of hospital where admitted अस्पताल का प्रकार जहाँ भर्ती की गई<br><br>(Government hospital सरकारी अस्पताल =1; Govt. Super-specialty hospital सरकारी विशेषज्ञता अस्पताल =2; Pvt. Nursing homes निजी नर्सिंग होम =3; Pvt /Corporate hospitals निजी/कॉरपोरेट अस्पताल =4; Pvt hospitals under EWS category ई.डब्ल्यू.एस.श्रेणी का निजी अस्पताल =5; RSBY-empanelled private hospital आर.एस.बी.वाई.सूचीबद्ध निजी अस्पताल =6; Others(specify) अन्य (स्पष्ट करें) =98; DK/CS=99)___________			
178	Name of the hospital अस्पताल का नाम			
179	Type of the facility<br>सुविधा का प्रकार<br>*Govt. clinic/dispensary* सरकारी क्लिनीक/डिसपेंसरी *=01;*<br>*Govt hospital* सरकारी अस्पताल*=02;*<br>*Private physician/clinic* निजी चिकित्सक/क्लिनीक *=03;*<br>*Private hospital/nursing home (including NGO/trust hospitals)* निजी अस्पताल/नर्सिंग होम(एन.जी.ओ./ट्रस्ट अस्पतालों से सहित) *=04;*<br>*Quacks/informal providers* फर्जी चिकित्सक/असंगठित प्रदाता *=05;*<br>*Medicine shops/pharmacy/old prescriptions* दवाखाना/फार्मेसी/पूराने नुस्खे *=06;*<br>*Hakim/vaids* हकीम/वैद्य *=07;*<br>_________________________*Other* (specify) अन्य (स्पष्ट करें) =98)			
180	Type of ward वार्ड का प्रकार<br><br>free निःशुल्क =1; paying general सशुल्क सामान्य=2; paying special सशुल्क विशेष =3; *Others* (specify) अन्य (स्पष्ट करें) =98_______________			
181	Days of stay कितने दिनों तक भर्ती			

Please provide costs/expenses related to the hospitalization during last 12 months **[COMBINED FOR ALL MEMBERS]**
कृपया विगत 12 महीनों में अस्पताल में भर्ती से संबंधित लागत/खर्चों को के बारे में बताएं (घर के सभी सदस्यों के खर्चों का बताएं)

	Please provide amount spent for<br>कृपया खर्च की गए राशि के बारे में बताएं<br>**For Other Hospitalizations**<br>**अन्य अस्पताल में भर्ती**<br>**Col (1)**	Total cost<br>कुल लागत<br>**Col (2)**	Amount paid out-of pocket (own income/savings)<br>दी गई राशि (अपनी आय/बचत)<br>**Col (3)**	Amount from other sources<br>अन्य स्रोतों से राशि<br>**Col (4)**	Source<br>स्रोत<br>**Col (5)**	Amount provided under RSBY scheme<br>आर.एस.बी.वाई.योजना के तहत दी गई राशि<br>**Col (6)**
182	Doctor's fee चिकित्सक का शुल्क					
183	Diagnostic tests निदान के लिए जाँच					
184	Medicine/injections दवाई/सूईयाँ					
185	Bed charges बेड शूल्क					
186	Surgery charges शल्य चिकित्सा शुल्क					
187	Any special diet/other food<br>कोई विशेष आहार/अन्य भोजन					
188	Follow-up costs<br>आगे की जाँच की लागत					
189	Transportation (including ambulance)<br>परिवहन (एम्बुलेंश सहित)					
190	Any other indirect costs (wage loss of attendants etc.) कोई और अप्रत्यक्ष खर्च (सेवक को पारिश्रमिक का नुकसान आदि)					
191	**Total expenses**<br>कुल खर्च					

Instructions: निर्देश
For 3: Ask respondent whether entire amount was met with self-income/salary/from other household member. If so, copy the total costs from Col.2 to Col. 3 and skip col.4 & col.5 उत्तरदाता से पूछें कि क्या पूरी राशि खुद की आय/वेतन/घर के अन्य सदस्य से पूरी की गई। यदि हाँ, कॉलम 4 एवं कॉलम 5 को छोड़ कॉलम 2 से कॉलम 3 में लिखित कुल खर्चे को नकल कर लें।
For 4: Entre the amount that was with supported from other sources: अन्य स्रोतों से प्राप्त कुल राशि को प्रविष्ट कर लें :
If Partial support=include amount यदि आंशिक मदद=राशि शामिल करें
If total (entire amount supported by external sources)=copy Col. 2. यदि योग (वाह्य स्रोत से प्राप्त कुल राशि)=कालम 2 की नकल कर लें
For 5: Other/External sources: अन्य/बाह्य स्रोत :
1=Borrow from friend/neighbor/relatives; मित्रों/पड़ोसियों/रिश्तेदारों से कर्ज
2=Loans from office; दफतर से उधार
3=Paid by the employee कर्मचारी द्वारा भुगतान
4=Loan (with interest) from money lender साहूकार से लिया गया सूद के साथ उधार
9=Others (specific) अन्य (स्पष्ट करें)

192	How were the expenses met? (*Multiple responses possible) Note: These questions are to be asked if the respondent is unable to give break-up of the expenses incurred (and its financing sources)* ***(Multiple responses possible)***<br><br>खर्चे कैसे पूरे किये गए ? **(अनेक उत्तर संभव हैं)**<br>नोट : ये प्रश्न तब पूछे जाने है *यदि उत्तरदाता खर्च (उसके वित्तीय स्रोत)टुकड़ों में नहीं बता पता है*	*Self savings* खुद की बचत	1
		Employer paid नियोक्ता द्वारा देय	2
		Friends/family मित्रों/रिश्तेदारों	3
		Health ins-RSBY स्वास्थ्य में आर.एस.बी.वाई	4
		Health insurance स्वास्थ्य बीमा *(others* अन्य*)*	5
		Borrowings from money lenders with interest साहूकारों से सूद सहित लिया गया कर्ज	6
		Selling of assets संपत्ति बेचकर	7
		Others (specify) अन्य	98
		DK/CS	99
193	Was it of any difficulty for your family/yourself to meet the costs associated with your (child's) treatment, and spending the amount you mentioned?<br>क्या आपके/आपके परिवार के लिए आपके बच्चे के उपचार पर खर्च, जिसके बारे में आपने उल्लेख किया, करने में किसी तरह की कठिनाई हुई ?	1	*Extremely difficult* अत्याधिक कठिनाई
		2	*Somewhat difficult* कुछ हद तक कठिनाई
		3	*Not at all* बिलकुल नहीं

Group C: No card + No Use
खंड स : कार्ड नहीं + इस्तेमाल नहीं

Now, I would like to ask you a few questions regarding your Knowledge with the RSBY scheme.

अब मैं आप से **RSBY (आर.एस.बी.वाई.)** योजना से संबंधित कुछ प्रश्न पूछना चाहूँगा ?

C1	Are you aware of a government scheme/programme that covers hospitalization expenses for families such as yours? क्या आपने, आपके ही समान, परिवारों के लिए भर्ती पर देय खर्चों से संबंधित सरकार की एक योजना/कार्यक्रम के बारे में सुना है ?	**Yes (1)**	**No (2)**
C2	Have you ever heard of a scheme called Rashtriya Swasthys Bima Yojana (RSBY)? क्या आपने राष्ट्रीय स्वास्थ्य बीमा योजना(आर.एस.बी.वाई.) नामक योजना के बारे में सुना है ?	**Yes (1)**	**No (2)**
C3	Have you ever seen any of your neighbours/relatives/friend having a card such as this? [SHOW RSBY CARD] क्या आपने अपने किन्हीं पड़ोसियों/रिश्तेदारों/मित्रों के पास इस तरह(आर.एस.बी.वाई. कार्ड दिखाएं) का कार्ड देखा है ?	**Yes (1)**	**No (2)**
**IF ANSWER "NO" to all of the 3 questions above↑, Skip to next Section** यदि उपरोक्त तीनों प्रश्नों का उत्तर नहीं है, तो अगले भाग में जाएं→ **IF ANSWER "YES" to any of the 3 questions above go to next question** यदि उपरोक्त तीनों प्रश्नों में किसी भी एक प्रश्न का उत्तर हाँ है, तो अगले प्रश्न पर जाएं ↓			
C4	Are you aware of the benefits received under RSBY scheme क्या आप आर.एस.बी.वाई.योजना के तहत प्राप्त लाभों के बारे में अवगत हैं।	**Yes (1)**	**No (2)**
C5	Why have you/your family not enrolled in the programme/RSBY? आप/आपका परिवार इस कार्यक्रम/आर.एस.बी.वाई. के तहत नामांकित क्यों नहीं है<br>Unsure about enrolment process; नामांकन प्रक्रिया के बारे में आश्वस्त नहीं हैं=1<br>Do not have required documents; वांछित दस्तावेज नहीं है=2<br>Lack of time to visit enrolment centre/get enrolled नामांकन कराने/नामांकन केन्द्र पर जाने के लिए समय का अभाव=3<br>Was refused enrolment; नामांकन के लिए मना कर दिया गया=4<br>Was told that the scheme is no longer operational; बताया गया की अब यह योजना चलन में नहीं है.=5<br>Unaware about the actual benefit; वास्तविक लाभ से अनभिज्ञ=6<br>Considers that the benefit will be too little मत यह है कि मिलने वाला लाभ बहुत कम है.=7<br>Unsatisfactory experience of friends/neighbours मित्रों/रिश्तेदारों का असंतोषजनक अनुभव=8<br>98=Others (Specify) कोई दूसरा (स्पष्ट करें)<br>99=Can't say/DK नहीं बता सकता/नहीं जानता		
C6	Do you consider that it will be useful/helpful for your family if you are enrolled under RSBY/and can avail of the facilities/financial support it provided? Would you say it will be: क्या आप मानते हैं कि आर.एस.बी.वाई के तहत नामांकन कराना आपके परिवार के लिए उपयोगी/सहायक होगा और आपका परिवार इसके तहत मिलने वाली सुविधाओं/वित्तीय मदद का लाभ ले सकेगा ? क्या आप कहेंगें कि ऐसा होगा :	Very useful बहुत उपयोगी	1
		Somewhat useful कुछहद तक उपयोगी	2
		Not useful at all बिलकुल उपयोगी नहीं	3
C7	For families such as yours, what kind of help/support do you think the government should provide, with regards to healthcare/medical treatment needs of your family and the financial implications associated with it? **(OPEN ENDED QUESTION/Note down)** आप के जैसे परिवारों के लिए, स्वास्थ्य सुविधाओं/चिकित्सकीय उपचार और वित्तीय निहितार्थ आपके अनुसार सरकार को किस प्रकार की मदद/सहायता का प्रावधान करनी चाहिए ?		

SECTION 4: WELFARE IMPACTS OF HEALTH SHOCKS & COPING MECHANSIMS

स्वास्थ्य सदमों एवं उसका सामना करने के तरीकों का कल्याणकारी प्रभाव

194	Considering all events of diseases/illnesses, hospitalizations, accidents, injury experienced by your family members in last 1 year, how would you rate the impact of all these events taken together, on the economic status/capacity of your household?<br>पिछले 1 वर्ष में आपके परिवार के सदस्यों द्वारा अनुभूत रोगों/बीमारियों, अस्पताल में भर्ती, दुर्घटनाओं, चोट को एकसाथ लेते हुए उनका आपके घर की आर्थिक स्थिति/क्षमता पर पड़ने वाले प्रभावों का आप कैसे मुल्यांकन करेंगे ?	Very severe impact<br>बहुत गंभीर प्रभाव	1
		Sever impact<br>गंभीर प्रभाव	2
		Moderate impact<br>औसत प्रभाव	3
		Partial impact<br>आंशिक प्रभाव	4
		No effect<br>कोई प्रभाव नहीं	5
195	Do you consider that your family has fully recovered, partially recovered, or not at all recovered from the financial impacts of the health shocks - taking all of these adverse health events together?<br>सभी विपरित स्वास्थ्य संबंधी घटनाओं पर एक साथ विचार करते हुए क्या आप मानते है कि आपका परिवार स्वास्थ्य सदमों के वित्तीय कुप्रभावों से पूरी तरह, आंशिक रूप से या बिलकुल ही नहीं उबर सका है ?	*Completely recovered*<br>पूर्णतः आरोग्य	1
		Partially recovered<br>आंशिक रूप से आरोग्य	2
		Not at all recovered<br>बिलकुल आरोग्य नहीं	3
196	Do you consider for some families, health shocks/adverse health events have been seen to be responsible for making families poorer, and impoverished in some cases.<br>Now, considering the economic status of your household during same time of last year (March, 2013/ Last Holi) and the health shocks/adverse health events your family has faced since then, do you think that the economic status/capacity of your household have:<br>क्या आप मानते हैं कि कुछ परिवारों को स्वास्थ्य संबंधी सदमों/खराब स्वास्थ्य की घटनाओं ने परिवार को और भी गरीब एवं बदहाल बना दिया है ?<br>अब आप बताएं कि पिछले साल की इसी अवधि (मार्च 2013/पिछली होली) में आपके परिवार की आर्थिक माली हालात की तुलना में ,परिवार में घटित स्वास्थ्य सदमों/खराब स्वास्थ्य की घटनाओं के बाद से, क्या आप सोचते हैं कि आपके परिवार की आर्थिक स्थिति/क्षमता में आज .... हुआ है ?	*Deteriorated considerably*<br>अत्याधिक गिरावट	1
		Deteriorate somewhat<br>कुछ हद तक गिरावट	2
		More or less remained the same<br>कुलमिलाकर यथास्थिति	3
		Improved somewhat<br>कुछ हदतक सुधार	4
		Substantially improved<br>ठोस सुधार	5

Do you think that your household/any family member had to do any of the following to cope with/manage the financial impacts arising out of all these health events taken together?
क्या आप सोचते हैं कि आपके घर/घर के किसी सदस्य को निम्नांकित में कोई भी कदम सभी स्वास्थ्य घटनाओं से जनित वित्तीय प्रभावों का सामना/प्रबंधन करने के लिए उठाना पड़ा था ?
(*1=Severe impact* गंभीर प्रभाव *, 2=High, but not severe impact* बड़ा किन्तु गंभीर नहीं *, 3=Somewhat/little impact* कुछ हदतक/थोड़ा प्रभाव *, 4=No impact* कोई प्रभाव नहीं)

197	Postpone marriages in the family<br>परिवार में विवाह आयोजन को लंबित करना पड़ा	1	2	3	4
198	Withdraw children from schools<br>बच्चों को स्कूल छुड़वाना पड़ा	1	2	3	4
199	Children started to work<br>बच्चों को काम पर लगाना पड़ा	1	2	3	4
200	Elderly members started to work<br>बुजर्गों को काम पर जाना पड़ा	1	2	3	4
201	Adults forced to take up additional working hours<br>व्यस्कों को काम पर अतिरिक्त समय देने के लिए बाध्य होना पड़ा	1	2	3	4
202	Reduce food consumption (e.g. adults skipping meals, buy cheaper/less quality food)<br>भोजन की खुराक कम करनी पड़ी (जैसे व्यस्कों को भोजन छोड़ना पड़ा, सस्ते/कम गुणवत्ता वाले भोजन खरीदना पड़ा)	1	2	3	4

203	Reduce other non-food consumption expenditure (on clothing, festival-spending, travel to native places, entertainment etc)<br>उपभोग के गैर–खाद्य वस्तुओं पर खर्चा कम करना (कपड़े पर, उत्सवों पर, गाँव घर जाने पर, मनोनंजन आदि)	**1**	**2**	**3**	**4**
204	Postpone/defer purchase of assets<br>संपत्ति की खरीद को लंबित करना/टालना	**1**	**2**	**3**	**4**
205	Delay/Ignore/Avoid non-critical health care needs of household members<br>घर के सदस्यों की छोटी स्वास्थ्य संबंधी जरूरतों में देरी करना/को नजरअंदाज करना/से बचना	**1**	**2**	**3**	**4**

206	Would you be able to raise Rs. 10,000 in one week if you needed it for some emergency?<br>किसी आपात की स्थिति में क्या आपके लिए एक सप्ताह के अंदर 10 हजार रूपया की व्यवस्था कर पाना संभव है ?	**Yes (1)**	**No (0)**	**DK/CS (99)**

207	How would you raise Rs. 10,000? आप 10,000 रूपयों की व्यवस्था कैसे करेंगे ?<br><br><u>Mention top 3 sources in the order you would follow</u><br><br>निम्नांकित क्रम में दिए गए स्रोतों में शीर्ष 3 स्रोतों का उल्लेख करें<br><br>(*Selling durable goods/equipment* लंबे समय तक चलने वाली वस्तुओं/उपकरणों को बेचकर *=1, Selling land/house* जमीन/घर बेचकर *=2 , From savings* बचत के पैसों से *=3, From relatives/friends in Delhi* दिल्ली के अपने मित्रों/रिश्तेदारों से *=4, From relatives/friends in other places* अन्य स्थानों पर रह रहे अपने रिश्तेदारों/मित्रों से *=5, From employer* नियोक्ता से *=6, From moneylender* साहूकारों से *=7, Taking extra work* अतिरिक्त काम कर *=8, From any MFI/cooperative/NBFI* किसी भी एम.एफ.आई./सहकारी समिति/एन.बी.एफ.आई. से *=9; Others* (specify) अ =98 अन्य(स्पष्ट करें); *DK/CS=99*)	**1st**	**1**	**2**	**3**	**4**	**5**	**6**	**7**	**8**	**9**	**99**
		**2nd**	**1**	**2**	**3**	**4**	**5**	**6**	**7**	**8**	**9**	**99**
		**3rd**	**1**	**2**	**3**	**4**	**5**	**6**	**7**	**8**	**9**	**99**

SECTION 5: QUALITY OF MEDICAL SERVICES/QUALITY OF CARE

भाग 7 : चिकित्सकीय सेवाओं की गुणवत्ता/देखभाल की गुणवत्ता

208		(a) Have you visited/accompanied patient to any health provider/ hospital/clinic for out-patient care (OPD) in last 12 months पिछले 12 महीनों के अंदर क्या आप किसी रोगी के साथ किसी स्वास्थ्य प्रदाता/अस्पताल/क्लिनीक में गए ?		(b)Have you been hospitalized/spent time with other family members **during hospitalization** in- patient care in last 12 months पिछले 12 महीनों में क्या आपकी भर्ती हुई/आपने परिवार के अन्य सदस्यों जिनकी इलाज के लिए भर्ती करवाई गई, के साथ समय बिताया ?	
		Yes (1)<br>***If yes, complete the full column*** ↓<br>यदि हाँ, तो पूरे कॉलम को भरें	No (0)<br>***If no, move to (b)***<br>यदि नहीं, तो (b) में जाएं	Yes (1)<br>***If yes, complete the full column*** ↓<br>यदि हाँ, तो पूरे कॉलम को भरें	No (0)<br>***If no, Skip to next Sub-Section***<br>यदि नहीं, तो अगले भाग में जाएं
209	Type of health facility used प्रयुक्त स्वास्थ्य सुविधा का प्रकार	Government hospital सरकारी अस्पताल (1)		Government hospital सरकारी अस्पताल (1)	
		Private hospital/nursing/clinics निजी अस्पताल/नर्सिंग होम/क्लीनिक (2)		Private hospital/nursing/clinics निजी अस्पताल/नर्सिंग होम/क्लीनिक (2)	
		Private practitioner निजी चिकित्सक (3)		Private practitioner निजी चिकित्सक (3)	
		Informal providers/*Jhola chap etc.* अनौपचारिक प्रदाता/झोला छाप (4)		Informal providers/*Jhola chap etc.* अनौपचारिक प्रदाता/झोला छाप (4)	
210	Rank the top three services, which you liked/found best during your last visit to a hospital/ nursing home/clinic<br><br>जब आप अंतिम बार अस्पताल/नर्सिंगहोम/क्लीनिक गए तो उस दौरान की तीन शीर्ष सुविधाओं जिसे आपने पसंद किया/सर्वोत्तम पाया का मूल्यांकन करें।		**Rank (1,2,3)**	**Rank (1,2,3)**	
		Skill and Competency of the staff/physician कर्मियों/चिकित्सकों का कौशल एवं योग्यता			
		Overall Cleanliness कुलमिलाकर सफाई			
		Friendliness and courtesy of the staff<br>कर्मियों का मित्रतापूर्ण एवं शालीन व्यवहार			
		Effectiveness of the treatment and medicine prescribed उपचार एवं लिखी गई दवाईयों की प्रभावकारिता			
		Cost of the treatment उपचार की लागत			
		Ease of communicating treatment/ therapy आसानी से उपचार/रोगाचार का संप्रेषण			
		Any other aspects (Specify) कोई अन्य पहलू (स्पष्ट करें)			

			**Rank (1,2,3)**	**Rank (1,2,3)**
211	Rank the top three services, which you disliked/found worst during your last visit to a hospital/ nursing home/clinic<br><br>जब आप अंतिम बार अस्पताल/नर्सिंगहोम/क्लीनिक गए तो उस दौरान की तीन शीर्ष सुविधाओं जिसे आपने पसंद नहीं किया/घटिया पाया का मूल्यांकन करें।	Distance दूरी		
		Waiting Time इंतजार का समय		
		Cleanliness/ Environment साफ सफाई/माहौल		
		Lack of Privacy during consultation/ overcrowding परामर्श के दौरान निजता का अभाव/भीड़		
		Behaviour of staff/ physician कर्मी/चिकित्सक का व्यवहार		
		Cost of treatment उपचार की लागत		
		Effectiveness of the treatment and medicine prescribed उपचार एवं लिखी गई दवाईयों की प्रभावकारिता		
		Any other aspects (Specify) कोई अन्य पहलू (स्पष्ट करें)		

Now, I am going to ask you a few questions on the health services
You have received/experienced while you were at the hospital clinic and your overall experience.
अब मैं आप से स्वास्थ्य सेवाओं के संबंध में कुछ प्रश्न करना चाहूँगा
अस्पताल/क्लीनिक में आपने क्या पाया/अनुभव किया एवं आपका कुल मिलाकर अनुभव

	**Domains** क्षेत्र	**For out- patient care** वाह्य रोगियों के देखरेख के लिए **(OPD)**	**For in- patient care/hospitalization** अंत : / अस्पताल में भर्ती रोगियों के लिए
212	How approachable/friendly was the doctor?<br>डॉक्टर कितने मित्रवत थे ?<br>1=Very much बहूत अधिक; 2=somewhat कुछ हदतक; 3=not at all बिलकुल नहीं		
213	How approachable/friendly was the other staff (nurses/technicians/clerks)? अन्य कर्मियों (नर्स/तकनीकी कर्मी/लिपिक) कितने मित्रवत थे ?<br>1=Very much बहूत अधिक; 2=somewhat कुछ हदतक; 3=not at all बिलकुल नहीं		
214	Did you find the working hours convenient to you, given your normal daily tasks/duties?<br>अपने दैनिक जिम्मेवारियों/दायित्वों के बीच क्या आपने कार्य अवधि को अनुकूल पाया ?<br>1=Very convenient बहुत अनुकूल; 2=Somewhat convenient कुछ हदतक अनुकूल; 3=Inconvenient प्रतिकुल; 4=Very inconvenient बहुत असुविधाजनक		
215	How convenient/accessible was the location of the facility/clinic from your residence?<br>आपके घर से सुविधा केन्द्र/क्लीनिक का स्थल कितना सुगम/सुविधाजनक था ?<br>1=Very convenient बहुत अनुकूल; 2=Somewhat convenient कुछ हदतक अनुकूल; 3=Inconvenient प्रतिकुल; 4=Very inconvenient बहुत असुविधाजनक		
216	Would you consider the time taken by the doctor/nurses/assistants properly explained to you the diagnosis and the treatment required?<br>क्या आपको लगता है कि जाँच एवं उपचार के लिए डॉक्टर/नर्स/सहायकों द्वारा दिया गया समय प्रर्याप्त था ? 1=Adequate प्रर्याप्त; 2= Somewhat Adequate कुछ हदतक प्रर्याप्त; 3= Inadequate अप्रर्याप्त		

217	Do you think that the doctor/nurses/assistants properly explained to you the diagnosis and the treatment required?<br><br>क्या आप सोचते हैं कि जरूरी जाँच एवं उपचार के बारे में डॉक्टर/नर्स/सहायकों ने आपको उचित तरीके से समझााया ? 1=Yes, very much हाँ, बहुत अच्छे से; 2=Somewhat normally explained कुछ हदतक सामान्य तरीके से; 3=Not at all बिलकुल नहीं		
218	In case you have had any queries regarding your/patient's problems/treatment how were it responded? Would you the response were:<br><br>जब आपने अपने/अपने रोगी के समस्याओं/उपचार के बारे में कोई पूछताछ की तो आपको किस तरह जबाव दिया गया ? क्या आप कहेंगें कि जबाव ...... था ?<br><br>1=Polite & adequate नम्र एवं पर्याप्त; 2=Polite but inadequate नम्र किन्तु अपर्याप्त; 3=Rude & inadequate अभद्र एवं अपर्याप्त		
219	How would you rate the privacy available, when the doctor was examining you/the patient?<br>जब डॉक्टर आपकी/आपके रोगी की जाँच कर रहे थे, तो जाँच में निजता का आप कैसे मूल्यांकन करेंगें ?<br>1=Satisfied संतुष्ट /good अच्छा ; 2=Somewhat satisfied कुछ हदतक संतुष्ट ; 3=Poor/not satisfied खराब/असंतुष्ट		
220	How would you rate the facilities available for emergency care? मौजूद आपातकालीन सेवाओं और सुविधाओं का आप कैसे मूल्यांकन करेंगे ?<br>1=Satisfied संतुष्ट /good अच्छा ; 2=Somewhat satisfied कुछ हदतक संतुष्ट ; 3=Poor/not satisfied खराब/असंतुष्ट		
221	How would you rate the – availability of drugs and medicines? दवाईयों की उपलब्धता का मुल्यांकन आप कैसे करेंगें ?<br>1=Satisfied संतुष्ट /good अच्छा ; 2=Somewhat satisfied कुछ हदतक संतुष्ट ; 3=Poor/not satisfied खराब/असंतुष्ट		
222	How would you rate the – hygiene, sanitation, and cleanness at the facility सुविधा केंद्र पर स्वास्थ्य, स्वच्छता एवं सफाई का आप कैसे मूल्यांकन करेंगे ?<br>1=Satisfied संतुष्ट /good अच्छा ; 2=Somewhat satisfied कुछ हदतक संतुष्ट ; 3=Poor/not satisfied खराब/असंतुष्ट		
223	How confident were you in the treatment that was being provided? आपको जो उपचार दिया जा रहा था उसपर आपको कितना विश्वास था ?<br><br>1=Very confident बहुत आश्वस्त ; 2=Somewhat confident कुछ हदतक आश्वस्त; 3=Not at all confident बिलकुल आश्वस्त नहीं		
224	How would you rate the effectiveness of the treatment provided? आप दिए गए उपचार की प्रभावकारिता का मूल्यांकन कैसे करेंगे ?<br>1=Very effective बहुत प्रभावकारी; 2=Somewhat effective कुछ हदतक प्रभावकारी; 3=Ineffective प्रभावहीन		
225	In case you had to wait for your turn, while seeking care (being treated at the facility (for consultation, tests or obtaining drugs etc.), how proper would you consider the waiting time was? यदि उपचार केन्द्र पर उपचार(परामर्श के लिए, जाँच या दवाईयाँ आदि) के लिए आपको अपनी बारी का इंतजार था, इंतजार का समय आपके ख्याल से कितना उचित था ?<br>1=Proper/OK उचित/ठीक; 2=Somewhat proper/manageable कुछ हदतक उचित/सहनीय; 3=Very long/frustrating बहुत लंबा/निराशाजनक		
226	Do you feel that you/patient were kept in the hospital longer than required?<br>क्या आपको ऐसा महसूस हुआ कि आप/आपके रोगी को अस्पताल में आवश्यकता से अधिक समय तक रोका गया ?<br>1=Yes; 0=No		

227	During the stay, how convenient would you consider was the facilities for having your friend/relatives etc. visit/interact/accompany you?<br>रूकने के दौरान आपके मित्रों/रिश्तेदारों के लिए क्या वहाँ पर्याप्त सुविधाएं थी ?<br>1=Very convenient बहुत अनुकूल; 2=Somewhat convenient कुछ हदतक अनुकूल; 3=Inconvenient प्रतिकुल; 4=Very inconvenient बहुत असुविधाजनक		
228	How would you rate the safety/security at the hospital?<br>अस्पताल में सुरक्षा का मूल्यांकन आप कैसे करेंगे ?<br>1=Satisfied संतुष्ट /good अच्छा ; 2=Somewhat satisfied कुछ हदतक संतुष्ट ; 3=Poor/not satisfied खराब/असंतुष्ट		
229	Were there only pressure/expectations/request for making any unofficial payment/bribes? क्या किसी प्रकार के गैरआधिकारिक भुगतान/घूंस के लिए दबाव/अपेक्षा/अनुरोध किया गया था ?<br>1=Yes; 0=No		

SECTION 6: INCOME & LIVELIHOOD [INFORMATION FOR ENTIRE HOUSEHOLD]

भाग 2 : आय एवं जीविका

230	What is the major income-earning activity of this household?<br>*(Formal sector employment/salaried service=1, Business/petty-trade=2, Self-employment=3, Manual/Wage-labour=4, Traditional semi-skilled trade=5, Remittance/Doles=6, Others* (specify)*=98)*<br>इस घर की आय अर्जित करने की मुख्य गतिविधि/किया क्या है ?<br>(संगठित क्षेत्र में रोजगार/वेतन आधारित सेवा=1, वाणिज्य/छोटा व्यापार=2, स्व–रोजगार=3, शारीरिक/वेतन भोगी मजदूर=4, पारंपरिक अर्द्ध कुशल व्यापार=5, प्रेषित रकम/बेरोजगारी भत्ता=6, अन्य (स्पष्ट करें)=98)			
**Do the household/any members of the household own the following?<br>क्या घर में/घर के किसी सदस्य के पास इनमें से कोई सामान है ?**				
231	Mobile phone मोबाइल फोन	**Yes (1)**	**No (0)**	**DK/CS (99)**
232	Motorcycles/scooters मोटर साईकल/स्कूटर	**Yes (1)**	**No (0)**	**DK/CS (99)**
233	Cycle साईकल	**Yes (1)**	**No (0)**	**DK/CS (99)**
234	Coolers कूलर	**Yes (1)**	**No (0)**	**DK/CS (99)**
235	Television टेलीविजन	**Yes (1)**	**No (0)**	**DK/CS (99)**
236	Television with cable/satellite TV connection<br>केबुल के साथ टेलीविजन/सेटेलाइट टेलीविजन कनेक्शन	**Yes (1)**	**No (0)**	**DK/CS (99)**
237	Any land (in Delhi/native place) कोई जमीन (दिल्ली में/पैत्तृक स्थान पर)	**Yes (1)**	**No (0)**	**DK/CS (99)**
238	Thinking of all the income-earning individuals in this household, which category would you say the household falls, taking the total MONTHLY household income/earning?<br>घर के सभी कमाने वाले लोगों को ध्यान में रखते हुए धर की कुल मासिक आय को देखते हुए आप अपने धर को किस श्रेणी में रखेंगे।	>5000	**1**	
		5000-7500	**2**	
		7500-10000	**3**	
		10000-15000	**4**	
		15000-30000	**5**	
		30000-50000	**6**	
		50000 & above	**7**	
		DK/CS	**99**	

A. Consumption expenditure उपभोग खर्च

**How much money was spent by your household – on<br>आपके घर में किस चीज पर कितना पैसा खर्च किया गया ?**		
239	(a) Food – during last 7 days (खाने पर – **पिछले 7 दिनों में**)	
240	(b) Electricity/Water Supply – during last 30 days (बिजली/जलापूर्ती पर – **पिछले 30 दिनों में)**	
241	(c) Transportation (to school/works) – during last 30 days<br>(परिवहन पर (स्कूल/कार्य स्थल जाने में) – **पिछले 30 दिनों में**)	
242	(d) Education of children (including tuition) – during last 30 days<br>बच्चों की शिक्षा (ट्यूशन को लेकर)पर – **पिछले 30 दिनों में**	

243	(e) Medical care/treatment (all diseases/injuries/by staying illness etc.) during last 30 days<br>पिछले 30 दिनों में चिकित्सकीय देखभाल/उपचार (सभी प्रकार के रोगों/चोटों/रोगों के रोकथाम आदि)	
244	(f) Rent/Taxes (for housing) – during last 30 days **पिछले 30 दिनों में** किराया/कर (धर के लिए)	
245	(g) Clothing/entertainment (movies/cable rent)/communications(mobile)/travels – during last 30 days **पिछले 30 दिनों में** कपड़ों/मनोरंजन (सिनेमा/केबल शुल्क)/संचार (मोबाइल)/यात्रा पर	
246	(h) Domestic durables (TV/bicycle/furniture) – during last 1year<br>लंबे समय तक इस्तेमाल होने वाले घरेलू सामान (टेलीविजन/साईकल/फर्नीचर) – **पिछले 1 वर्ष से**	
247	(i) Home repair/constructions – during last 1year<br>धर की मरम्मत/निर्माण – **पिछले 1 वर्ष में**	
248	(j) Clothing/footwear – during last 1year<br>कपड़े/जूते–चप्पल – **पिछले 1 वर्ष में**	
249	(k) Any other expenses – during last 1year<br>अन्य दूसरे खर्च – **पिछले 1 वर्ष में**	
250	(l) TOTAL कुल खर्च	

B. Nutrition and Diet पोषण एवं भोजन

How often are the following items eaten in your household?

निम्नलिखित चीजें आपके घर में कितनी बार खाई जाती हैं ?

(1=Daily रोज
2=For most days of the week सप्ताह के अधिकांश दिनए
3=Rarely/Occasionally कभी कभार/कभी कभी
9=Never कभी नहीं
98= Others (specify) अन्य (स्पष्ट करें)
99=DK/CS नहीं जानते)

251	Fruits फल	**1**	**2**	**3**	**9**	**98**	**99**
252	Green Vegetables हरी सब्जियाँ	**1**	**2**	**3**	**9**	**98**	**99**
253	Milk दूध	**1**	**2**	**3**	**9**	**98**	**99**
254	How often do you have food outside your home?<br>घर से बाहर खाना आप कितनी बार खाते हैं ?	**1**	**2**	**3**	**9**	**98**	**99**

C. Physical Activity (No proxy response) [ONLY FOR RESPONDENTS/ NO PROXY RESPONSE]

शारीरिक कार्य (प्रतिनिधि उत्तर नहीं)

For each of the type of activity mentioned below, please indicate how often you did these activities, during last 1 month?
पिछले 1 महीने के अंदर आपने न्मिांकित कार्य आपने कितनी बार किया ?

(1=Daily रोज
2=For most days of the week सप्ताह के अधिकांश दिनए
3=Rarely/Occasionally कभी कभार/कभी कभी
9=Never कभी नहीं
98= Others (specify) अन्य (स्पष्ट करें)
99=DK/CS नहीं जानते)

255	Heavy manual work (digging, lifting heavy-loads>5kg, pulling vans/rickshaw)<br>भारी शारीरिक कार्य (खोदना, भारी वनज उठाना 5किलो से अधिक, माल गाड़ी/रिक्शा खींचना	**1**	**2**	**3**	**9**	**98**	**99**
256	Other manual work/activity (carrying water, laundry, swimming, playing football अन्य शारीरिक श्रम/क्रिया (पानी की ढुलाई, कपड़े साफ करना, तैरना, फुटबाल खेलना	**1**	**2**	**3**	**9**	**98**	**99**
257	Ride bicycle for more than 1 hour/day प्रतिदिन एक घंटे से अधिक साईकिल की सवारी	**1**	**2**	**3**	**9**	**98**	**99**
258	Walk for at least 30 minutes/day प्रतिदिन कम से कम 30 मिनट टहलना	**1**	**2**	**3**	**9**	**98**	**99**

259	Routine domestic chores (cleaning utensils, cooking etc..)<br>नियमित घरेलू कार्य (बरतन साफ करना, रसोई का काम ....)	**1**	**2**	**3**	**9**	**98**	**99**
260	Other sedentary work (teaching, shop-keeping, artisans..)<br>अन्य बैठकर किए जाने वाले काम (शिक्षण,दुकानदारी, कारीगर ....)	**1**	**2**	**3**	**9**	**98**	**99**

D. Living Conditions रहन–सहन की स्थिति [INFORMATION FOR ENTIRE HOUSEHOLD]

261	*Location of the house/quarters* घर / क्वार्टर की जगह<br>*(Near Garbage dumps कचड़ों के ढेर के पास=1, On pavements पैदल चलने वाले रास्तों पर=2, Street-side गलियों के किनारे=3, Near/beside railway-lines रेल पटरियों के पास/किनारे=4, JJ Colonies जे जे कॉलोनियों में=5, Unauthorized colonies अनाधिकुत कॉलोनियों में=6, Resettlement colonies पुर्नवासित कॉलोनीयों में=7, Regularized colonies नियमित किए गए कॉलोनियों में=8, Others(specify) अन्य (स्पष्ट करें)=98)*	
262	Whether household members sleep in the same room where food is cooked<br>क्या घर के लोग उसी कमरे में सोते हैं जहाँ खाना पकाया जाता है ? *(Yes हाँ =1, No नहीं =0)*	
263	Type of toilet used by household members घर के लोगों द्वारा प्रयुक्त शौचालय का प्रकार<br>*(Open defecation खुले में शौच =1; Public toilet सार्वजनिक शौचालय =2; WC – in premises घेरे के अंदर =3; Flush फलश शौचालय-4.; Other (specify) अन्य (स्पष्ट करें)=98)*	
264	Main source of drinking water पीने के पानी का मुख्य स्रोत<br>*(Piped water पाईप से मिलने वाला पानी =1; Tubewell/handpump ट्यूबवेल/हाथ से चलाया जाने वाला नल =2; Public Tap सार्वजनिक नलकूप =3; Tanker टैंकर =4; Wells कुंआ =5; Other(specify) अन्य (स्पष्ट करें)=98)*	
265	If public tap water/tanker: Hours of availability<br>यदि सार्वजनिक नलकूप / टैंकर द्वारा : **उपलब्धता घंटों में**	______Hours (सार्वजनिक नलकूप)<br>_________No. of times (टैंकर)
266	Type of cooking fuel used रसोई के लिए प्रयुक्त ईंधन का प्रकार<br>*(Kerosene मिट्टी तेल =1; Coal/Cow-dung/other wastes कोयला/उपले/अन्य अवशेष =2; LPG=3; Electricity=4; Other (specify) अन्य (स्पष्ट करें)=98)*	
267	**Could you quote specific places where you often stop (from few minutes to hours) during the week (7 days):**	

**Purpose**	**Distance from your house (in meters and minutes)**		**Frequency (times per week)**	**Time**<br>*Morning – (1)*<br>*Midday/Noon - (2)*<br>*Afternoon – (3)*<br>*Evening/Nightfall - (4)*	**Mode of transportation**<br>*Cycle – (1)*<br>*On foot - (2)*<br>*Motorcycles – (3)*<br>*Public transport - (4)*<br>*Metro - (5)*
	**Meters**	**Minutes**			
a) Main Occupation/Place of Work					
b) Religious Places/ Places of Worship					
c) Shopping (Buying Supplies, Groceries, Vegetables)					
d) Visiting Relatives (friends and family)					

E. Mortality history

Screener: Were there any deaths in your family (include relatives/ancestral home) during the last 1 year? क्या पिछले 1 वर्ष के दौरान आपके परिवार(रिश्तेदारों/पैतृक गृह को शामिल करें) में किसी की मौत हुई ?	Yes (1)	No (0)	**If answers '0' End the interview**

[m1] Serial No. क्रम संख्या	[m2] Relationship with respondent उत्तरदाता के साथ संबंध	[m3] Age at the time of death मृत्यु के समय आयु	[m4] Sex लिंग	[m5] Highest education level उच्चतम शैक्षणिक स्तर	[m6] Activity/occupation गतिविधि/पेशा	[m7] Whether deceased suffered from any of the following diseases क्या मृत व्यक्ति को निम्नांकित में कोई रोग था ?	[m8] Cause of death मृत्यु का कारण	[m9] Place of death मृत्यु का स्थान	[m10] Medical care received before death मृत्यु के पूर्व प्राप्त चिकित्सकीय सुविधा	[m11] Type of medical institution चिकित्सा संस्थान का प्रकार	[m11a] Medical expenditure during hospitalization अस्पताल में भर्ती के दौरान खर्च	[m12] Whether consumed tobacco (smoke/smokeless) क्या तंबाकू का सेवन करते थे ?	[m12a] If yes, frequency of consumption* यदि हाँ, तो कितना	[m13] Whether consumed alcohol क्या शराब का सेवन करते थे?	[m13b] If yes, frequency of consumption* यदि हाँ तो कितनी बार	[m14] Whether death registered? क्या मृत्यु का निबंधन कियागया
MS01																
MS02																
MS03																
MS04																
MS05																
MS06																
MS07																

^^ Read out the diseases as in the Codebook and put the codes (multiple codes possible – Allow max 3). ^ Multiple codes possible – Allow max 3 *Record perceived/rough approximations – See Codebook कोड बुक के अनुसार रोगों को पढ़ें और कोड अंकित करें (अनेक कोड संभव हैं – अधिकतम 3)। अनेक कोड संभव हैं – अधिकतम 3 नजदीकी उत्तर के लिए कोड बुक देखें

CODES

*For m1: **Relation to head घर के मुखिया से संबंध** : Spouse=1, child=2, spouse of married child=3, unmarried child=4, grand child=5, father/mother/ father-in-law/mother-in-law=5, brother/sister/brother-in-law/sister-in-law/other relative=6, servant/employees/other non-relatives=7*

*For m4: **Sex लिंग**: male=1, female=2*

*For m5: **Educational level शैक्षणिक स्तर**: not literate=01; literate: without formal schooling=02, below primary=03, primary=04, Middle=05, secondary= 06, higher secondary= 07, diploma/certificate course=08, graduate=10, post-graduate & Above= 11*

*For m6: **Usual activity status सामान्य कार्य स्थिति** : Casual/daily wage laborer=01, Self-employed (including small business & trade)=02, Regular salaried employment=03, Unemployed/seeking work=04, Student=05, Home-maker/household work/domestic duties (unpaid)=06, Disabled/Old/Young=07, Household entrepreneur (tailoring/weaning/hand wash)=08, Others=99 (Specify)*

*For m7: **Decease suffered रोगों के शिकार :** Please see the attached additional sheet*

*For m8: **Cause of death मृत्यु का कारण :** Infections and parasitic diseases=1; Respiratory infections=2; Maternal conditions=3; Perinatal conditions=4; Nutritional deficiencies=5; Malignant neoplasms=6; Other neoplasms=7; Diabetes mellitus=8; Endocrine disorders=9; Neuropsychiatric conditions=10; Sense organ diseases=11; Cardiovascular diseases=12; Respiratory diseases=13; Digestive diseases=14; Genitourinary diseases=15; Skin diseases=16; Congenital anomalies=17; Unintentional injuries=18; Intentional injuries=19; Ill-defined injuries/accidents=20;*

*For m9: **Place of death मृत्यु का स्थान :** home=1, relatives home=2, hospital=3, on spot=4, in transit=5, others=6, DK/CS=99*

*For m10: **Medical care receive प्राप्त चिकित्सीय देखरेख :** Yes=1, No=2, DK/CS=99*

*For m11: **Type of medical institution चिकित्सकीय संस्थान का प्रकार** : 1=Govt. clinic/dispensary; 2=Govt hospital; 3=Private physician/clinic; 4=Private hospital/nursing home (including NGO/trust hospitals); 5=quacks/informal providers; 6=medicine shops/pharmacy; 7=hakim/vaids*

*For m12: **Consumed tobacco तंबाकू का सेवन** : smoking tobacco=1, smokeless tobacco=2, both=3, none=4*

For m13 & m14: Yes=1, No=2, DK/CS=99

**Respondent Mob. No.**										
