# Supplementary material for: Widespread fear of dengue transmission but poor practices of dengue prevention: A study in the slums of Delhi, India
Source: PLoS One. 2017 Feb 10;12(2):e0171543. doi: 10.1371/journal.pone.0171543 (PMC5302449; doi:10.1371/journal.pone.0171543)
Supplement: S2 File — (PDF) [file pone.0171543.s002.pdf]

ICMR-RSBY Sampling Frame

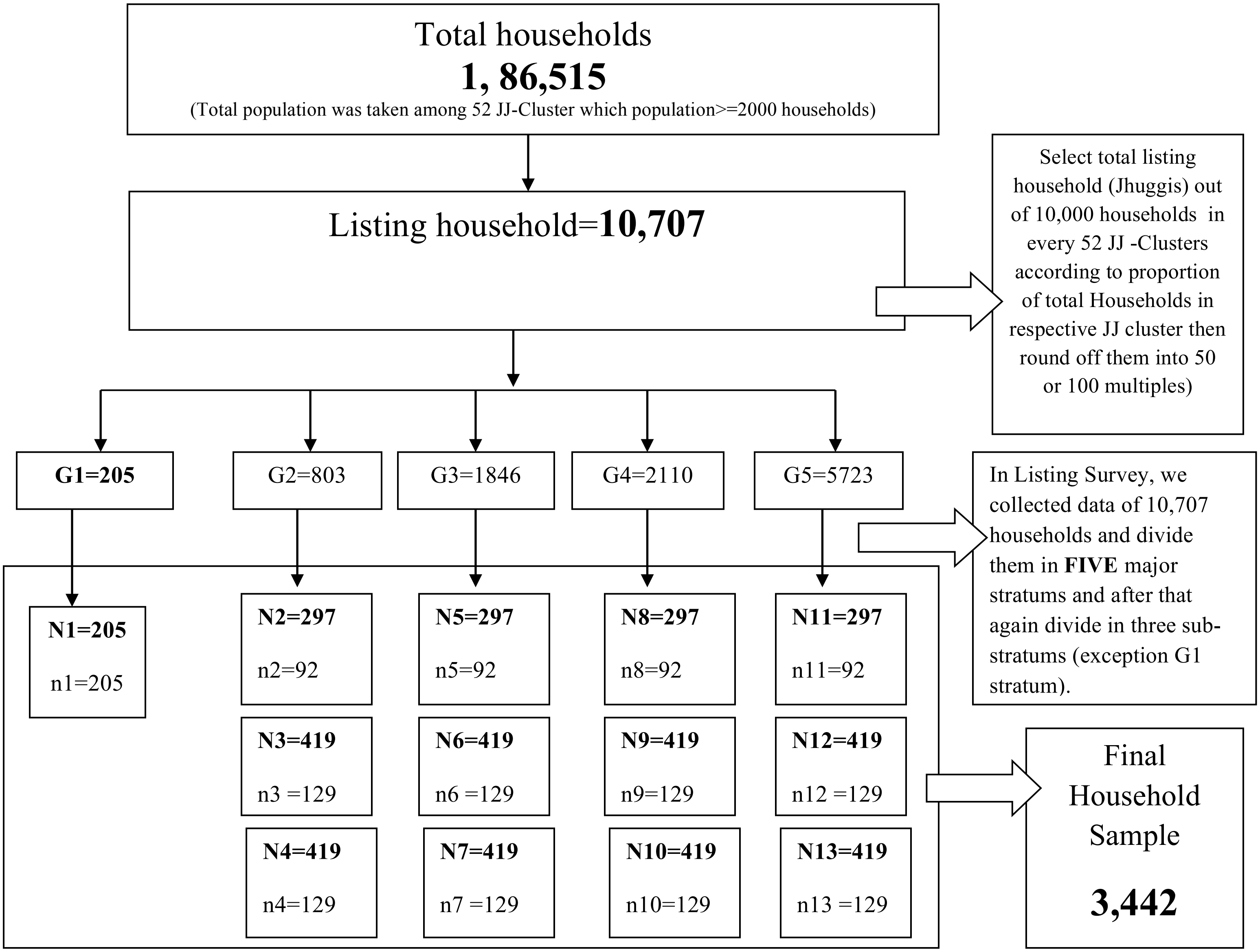

N=Listing population; n=household sample; SES=Socioeconomic Status

	Listing	Sample	
G1	N1	n1	Have RSBY Card+Hospitlised+Use Card
G2	N2	n2	Have RSBY Card+Hospitlised+Don't Use Card+SES1
	N3	n3	Have RSBY Card+Hospitlised+Don't Use Card+SES2
	N4	n4	Have RSBY Card+Hospitlised+Don't Use Card+SES3
G3	N5	n5	Have RSBY Card+Not Hospitlised+Don't Use Card+SES1
	N6	n6	Have RSBY Card+Not Hospitlised+Don't Use Card+SES2
	N7	n7	Have RSBY Card+Not Hospitlised+Don't Use Card+SES3
G4	N8	n8	No RSBY Card+Hospitalised+SES1
	N9	n9	No RSBY Card+Hospitalised+SES2
	N10	n10	No RSBY Card+Hospitalised+SES3
G5	N11	n11	No RSBY Card+Not Hospitalised+SES1
	N12	n12	No RSBY Card+Not Hospitalised+SES2
	N13	n13	No RSBY Card+Not Hospitalised+SES3
